# The Mistreatment of Women during Childbirth in Health Facilities Globally: A Mixed-Methods Systematic Review

**DOI:** 10.1371/journal.pmed.1001847

**Published:** 2015-06-30

**Authors:** Meghan A. Bohren, Joshua P. Vogel, Erin C. Hunter, Olha Lutsiv, Suprita K. Makh, João Paulo Souza, Carolina Aguiar, Fernando Saraiva Coneglian, Alex Luíz Araújo Diniz, Özge Tunçalp, Dena Javadi, Olufemi T. Oladapo, Rajat Khosla, Michelle J. Hindin, A. Metin Gülmezoglu

**Affiliations:** 1 Department of Population, Family and Reproductive Health, Johns Hopkins Bloomberg School of Public Health, Baltimore, Maryland, United States of America; 2 Department of Reproductive Health and Research including UNDP/UNFPA/UNICEF/WHO/World Bank Special Programme of Research, Development and Research Training in Human Reproduction, World Health Organization, Geneva, Switzerland; 3 Department of International Health, Johns Hopkins Bloomberg School of Public Health, Baltimore, Maryland, United States of America; 4 Department of Epidemiology, Biostatistics and Occupational Health, McGill University, Montreal, Quebec, Canada; 5 Population Services International, Washington, D. C., United States of America; 6 Department of Social Medicine, Ribeirão Preto Medical School, University of São Paulo, Ribeirão Preto, São Paulo, Brazil; Medical Research Council, SOUTH AFRICA

## Abstract

**Background:**

Despite growing recognition of neglectful, abusive, and disrespectful treatment of women during childbirth in health facilities, there is no consensus at a global level on how these occurrences are defined and measured. This mixed-methods systematic review aims to synthesize qualitative and quantitative evidence on the mistreatment of women during childbirth in health facilities to inform the development of an evidence-based typology of the phenomenon.

**Methods and Findings:**

We searched PubMed, CINAHL, and Embase databases and grey literature using a predetermined search strategy to identify qualitative, quantitative, and mixed-methods studies on the mistreatment of women during childbirth across all geographical and income-level settings. We used a thematic synthesis approach to synthesize the qualitative evidence and assessed the confidence in the qualitative review findings using the CERQual approach. In total, 65 studies were included from 34 countries. Qualitative findings were organized under seven domains: (1) physical abuse, (2) sexual abuse, (3) verbal abuse, (4) stigma and discrimination, (5) failure to meet professional standards of care, (6) poor rapport between women and providers, and (7) health system conditions and constraints. Due to high heterogeneity of the quantitative data, we were unable to conduct a meta-analysis; instead, we present descriptions of study characteristics, outcome measures, and results. Additional themes identified in the quantitative studies are integrated into the typology.

**Conclusions:**

This systematic review presents a comprehensive, evidence-based typology of the mistreatment of women during childbirth in health facilities, and demonstrates that mistreatment can occur at the level of interaction between the woman and provider, as well as through systemic failures at the health facility and health system levels. We propose this typology be adopted to describe the phenomenon and be used to develop measurement tools and inform future research, programs, and interventions.

## Abstract 2

Meghan Bohren and colleagues review the extent and types of mistreatment against women during childbirth.

## Editors' Summary

**Background:**

In 2000, as Millennium Development Goal (MDG) 5, world leaders set a target of reducing the global maternal mortality ratio—the number of deaths among women caused by pregnancy- or childbirth-related complications (maternal deaths) per 100,000 live births—to a quarter of its 1990 level by 2015. MDG 5, along with seven other MDGs, was designed to alleviate extreme poverty by 2015. Although progress towards MDG 5 (and towards the other MDGs) has been good, in 2013, the global maternal mortality ratio was still 210, well above the target of 95. In that year alone, nearly 300,000 women, 99% them living in low- and middle-income countries, died from pregnancy- or childbirth-related complications. Most of these maternal deaths were caused by hemorrhage (severe bleeding) after childbirth, post-delivery infections, obstructed (difficult) labor, or blood pressure disorders during pregnancy. These conditions are largely preventable if women have access to good-quality reproductive health services and if trained birth attendants are present during childbirth.

**Why Was This Study Done?:**

The rates of skilled birth attendance and of facility-based childbirth have risen in resource-limited countries over the past two decades, but almost a third of women in these countries still deliver without a skilled birth attendant. Among the numerous obstacles likely to prevent further increases in the proportion of women delivering in a health facility is women’s fear of mistreatment during delivery. Women need to be sure that they will receive dignified and respectful care during childbirth. Unfortunately, recent studies have indicated that women are often exposed to neglectful, abusive, and disrespectful care (care that local consensus regards as humiliating or undignified) during childbirth in health facilities. There is currently no consensus about how to define and measure the mistreatment of women during childbirth, so here, the researchers develop an evidence-based typology of the mistreatment of women during childbirth in health facilities worldwide by combining information identified in a mixed-methods systematic review. A typology is a systematic classification of objects or behaviors that have characteristics in common. A mixed-methods systematic review identifies all the qualitative and quantitative research on a given topic using predefined criteria. Qualitative research investigates how people feel about a medical intervention; quantitative research provides numerical data about interventions.

**What Did the Researchers Do and Find?:**

The researchers identified 65 (mainly qualitative) studies undertaken in 34 countries that investigated the mistreatment of women during childbirth across all geographical and income-level settings. They analyzed the evidence presented in these studies using thematic analysis, an approach that identifies and organizes patterns (themes) within qualitative data. Based on this analysis, the researchers developed a typology of the mistreatment of women during childbirth consisting of seven domains (categories). These domains were physical abuse (for example, slapping or pinching during delivery); sexual abuse; verbal abuse such as harsh or rude language; stigma and discrimination based on age, ethnicity, socioeconomic status, or medical conditions; failure to meet professional standards of care (for example, neglect during delivery); poor rapport between women and providers, including ineffective communication, lack of supportive care, and loss of autonomy; and health system conditions and constraints such as the lack of the resources needed to provide women with privacy.

**What Do These Findings Mean?:**

These findings illustrate how women’s experiences of childbirth worldwide are marred by mistreatment. Moreover, they indicate that, although the mistreatment of women during delivery in health facilities often occurs at the level of the interaction between women and healthcare providers, systemic failures at the levels of the health facility and the health system also contribute to its occurrence. Further studies are needed to provide quantitative evidence of the burden of mistreatment of women during delivery and to identify the characteristics of health facilities that facilitate or mitigate the mistreatment of women. For now, though, the researchers call for the adoption of their evidence-based typology as a way to describe the mistreatment of women during childbirth in health facilities. Their typology, they suggest, could also be used to develop measurement tools and to design interventions that ensure that health care providers promote positive birth experiences by providing respectful, dignified, and supportive care to women during childbirth. Hopefully, such interventions will lead to more women deciding to deliver their babies in health facilities, will promote positive birth experiences, and, ultimately, will lead to further reductions in maternal deaths.

**Additional Information:**

This list of resources contains links that can be accessed when viewing the PDF on a device or via the online version of the article at http://dx.doi.org/10.1371/journal.pmed.1001847.

- This study is further discussed in a *PLOS Medicine* Perspective by Rachel Jewkes and Loveday Penn-Kekana
- The World Health Organization (WHO) has published a statement on promoting the rights of women and access to safe, timely, respectful care during childbirth
- The White Ribbon Alliance promotes the universal rights of childbearing women
- The United Nations Children’s Fund (UNICEF) provides information on maternal health and on the recent decline in maternal mortality
- The World Health Organization provides information on maternal health, including information about Millennium Development Goal 5 (in several languages) and information about the prevention and elimination of disrespect and abuse during childbirth
- Further information about the Millennium Development Goals and *The Millennium Development Goals Report 2014*, which details progress on reaching these goals, are available (in several languages)
- Immpact is a research initiative for the evaluation of safe motherhood intervention strategies
- *Maternal Death*: *The Avoidable Crisis* is a briefing paper that was published by the humanitarian medical aid organization Médecins Sans Frontières in 2012
- Veil of Tears contains personal stories from Afghanistan about maternal death
- More information is available on the CERQual approach for assessing confidence in the evidence from reviews of qualitative research
- The Cochrane Qualitative and Implementation Methods Group has published supplemental handbook guidance on qualitative evidence syntheses
- Wikipedia has pages on thematic analysis and on systematic review (note that Wikipedia is a free online encyclopedia that anyone can edit; available in several languages)

## Introduction

An estimated 289,000 maternal deaths occurred in 2010, of which 99% occurred in low- and middle-income countries [1]. While maternal mortality has declined globally by 45% since 1990, progress towards Millennium Development Goal 5, a 75% reduction in the maternal mortality ratio between 1990 and 2015, has been slow, and many low- and middle-income countries will not reach this target [2]. Ensuring universal access to safe, acceptable, good quality sexual and reproductive health care, particularly contraceptive access and maternal health care, can dramatically reduce the global burden of maternal morbidity and mortality. A key component of the strategy to reduce maternal morbidity and mortality has been to increase rates of skilled birth attendance and facility-based childbirth. While global skilled birth attendance rates rose by 12% in developing regions over the past two decades, almost one-third of women in these regions still deliver without a skilled birth attendant [3]. Increasing the proportion of women delivering in a health facility is challenging, as it requires comprehensive efforts to overcome sociocultural, economic, geographical, and infrastructural obstacles to reaching facility-based care [4]. Furthermore, it requires efforts to improve both the coverage and quality of care provided to women at health facilities, including women’s rights to dignified and respectful care [5].

A recent qualitative evidence synthesis [4] and several recent studies [6–11] and reports [12,13] clearly indicate that many women globally experience poor treatment during childbirth, including abusive, neglectful, or disrespectful care. Every woman has the right to dignified, respectful sexual and reproductive health care, including during childbirth [14–16], as highlighted by the Universal Rights of Childbearing Women charter [16]. Therefore, mistreatment during childbirth can represent a violation of women’s fundamental human rights [17–20] and can serve as a powerful disincentive for women to seek care in facilities for their subsequent deliveries [4,6,10,21]. In September 2014, a World Health Organization statement called for greater research, action, advocacy and dialogue on this important public health issue, in order to ensure safe, timely, respectful care during childbirth for all women [5]. Likewise, respectful care is a key component of both the mother-baby friendly birthing facilities initiative [22] and the WHO vision for quality of care for childbearing women and newborns [23].

In a 2010 landscape analysis, Bowser and Hill described seven categories of disrespectful and abusive care during childbirth—physical abuse, non-consented clinical care, non-confidential care, non-dignified care, discrimination, abandonment, and detention in health facilities [12]—which have been a building block of recent work on this topic [24–27]. Freedman and colleagues built on the Bowser and Hill categories to propose a definition of disrespectful and abusive care during childbirth (e.g., to articulate criteria for determining when an interaction or condition should be considered abusive or disrespectful) and a conceptual model illustrating how this definition can vary across individual, structural, and policy levels [28,29]. Freedman and Kruk defined disrespect and abuse during childbirth as “interactions or facility conditions that local consensus deems to be humiliating or undignified, and those interactions or conditions that are experienced as or intended to be humiliating or undignified” [28].

The Bowser and Hill classification has some limitations. The authors integrated findings from a literature review and key stakeholder interviews to develop the seven categories; however, it appears that systematic searching and synthesis methodologies were not employed. Furthermore, three recent measurement studies based on the Bowser and Hill categories have used different operational definitions and study designs [25–27]. These variations may have contributed to the substantial differences in estimates of prevalence, preventing meaningful meta-analysis. The lack of standardized, comprehensive, and agreed typology, identification criteria, and operational definitions of the mistreatment of women during facility-based childbirth thus complicates further research in this important area.

Developing an evidence-based typology is therefore a critical step, in order to inform the development and application of measurement tools and to permit the development and evaluation of interventions to reduce mistreatment and promote respectful maternity care. Such efforts are necessary to improve the quality of maternity care, increase demand for facility-based childbirth, and, more broadly, protect women’s fundamental human rights. This systematic review aims to contribute to the development of a global evidence-based typology of the mistreatment of women during childbirth in health facilities.

## Methods

### Search Strategy

The published literature was systematically searched in PubMed (S2 Table), CINAHL (S3 Table), and Embase (S4 Table) using controlled vocabulary and free-text terms combining two main components: (a) maternal health, perinatal health, or childbirth and (b) mistreatment of women. Searches were conducted on 4 September 2013, and updated on 3 September 2014 and 11 February 2015, with no date or language restrictions. The WHO Global Health Library, Cochrane Library, Database of Abstracts of Reviews of Effects (DARE), Google Scholar, Centre for Reviews and Dissemination (CRD) Database, OpenGrey, and EThOS (British Library) were searched for grey literature and unpublished reports, and researchers in relevant fields were contacted for assistance in identifying studies. Reference lists of all included studies were hand-searched to identify additional studies.

### Study Selection

Each title and abstract was screened for inclusion by two independent reviewers using a standardized form (M. A. B., E. C. H., O. L., S. K., J. P. V.). Each full text article was reviewed by two independent reviewers using standardized inclusion criteria: (a) presents primary data analysis; (b) uses a qualitative method of data collection and analysis (qualitative studies); (c) discusses poor treatment of women during childbirth; (d) discusses childbirth occurring in health facilities; and (e) is published in English, French, Spanish, or Portuguese language (M. A. B., E. C. H., O. L., S. K., F. S., A. L. A. D., J. P. V., J. P. S.). Discrepancies during title and abstract and full text screening were resolved by discussion with a third reviewer until consensus was reached.

### Quality Assessment

The quality of the qualitative studies was assessed using an adaptation of the Critical Appraisal Skills Programme (CASP) quality-assessment tool (http://www.casp-uk.net), and assessment included the following domains: aims, methodology, design, recruitment, data collection, data analysis, reflexivity, ethical considerations, findings, and research contribution (M. A. B., E. C. H., O. L., C. A., J. P. V.). The quality of the quantitative studies was assessed using an adaptation of the STROBE (Strengthening the Reporting of Observational Studies in Epidemiology) statement (M. A. B., J. P. V.) [30], and assessment included the following domains: eligibility criteria, method of variable assessment, participant characteristics, reporting of summary measures/outcome events, and discussion of sources of bias and/or imprecision. The overall quality assessment of “high,” “medium,” or “low” was based on independent evaluation by two reviewers, with discussion until consensus was reached in the case of discrepancies. No studies were excluded as a result of the quality assessment; rather, the methodological rigor of each contributing study contributed to the confidence assessments of each review finding.

### Data Extraction

Data were extracted using a standardized form including the following domains: study setting, sample characteristics, objectives, design, data collection and analysis methods, and conclusions (M. A. B., E. C. H., O. L., S. K., F. S., A. L. A. D., J. P. S., J. P. V.). Themes, findings, and participant quotations were extracted from qualitative studies. Data source, outcome measures, and results were extracted from quantitative studies.

### Synthesis

#### Quantitative

We planned to present prevalence estimates of the mistreatment of women. However, meta-analysis was not possible due to high heterogeneity in the quantitative studies, including inconsistent identification criteria and operational definitions. Therefore, descriptions of study characteristics, outcome measures, and key findings are presented (M. A. B., O. L., J. P. V.).

#### Qualitative

A thematic synthesis approach was used to analyze and synthesize the qualitative data [31]. A spreadsheet was created of all qualitative data extracted from the studies’ findings sections, and thematic analysis methods were used to conduct initial open coding on each relevant text unit (M. A. B., E. C. H., J. P. V.) [32]. Based on the initial coding, 14 broad themes were developed, and all text units were iteratively classified into one of the broad themes. Each theme was further analyzed to inductively develop the axial coding scheme and to disaggregate core themes [33,34]. Axial codes were then systematically applied by hand-sorting the text units into first-, second-, and third-order themes (M. A. B., E. C. H., J. P. V.). First-order themes represent text units grouped together based on common descriptive themes. Second-order themes represent first-order themes grouped together based on higher-level analytic themes. Third-order themes represent overarching high-level analytic themes comprising the first- and second-level themes [35].

Each qualitative review finding was assessed using the CERQual (Confidence in the Evidence from Reviews of Qualitative Research) approach. CERQual is a method to transparently assess and describe how much confidence to place in findings from systematic reviews of qualitative evidence [36,37]. Our “confidence” is an assessment of the extent to which a review finding is a reasonable representation of the phenomenon of interest, such that the phenomenon of interest is unlikely to be substantially different from the research finding [36,37]. The CERQual approach is under development and currently includes four elements: (1) methodological limitations of the individual studies, (2) relevance to the review question, (3) coherence, and (4) adequacy of data, which we operationalized in the following manner (M. A. B., J. P. V.) [4,36–42]. The methodological limitations of the individual studies contributing to each review finding were assessed using the modified CASP tool. The relevance to the review question of the individual studies contributing to a review finding was assessed based on the extent to which the review finding was applicable to the context (perspective, population, phenomenon of interest, setting) specified in the review question. The coherence of each review finding was assessed by exploring to what extent clear patterns could be identified across the data contributed by each study, or whether plausible explanations were provided if variation across studies existed [38]. The adequacy of the data that supported a review finding was assessed in terms of the thickness of data, the number of studies, the stratification of countries/regions, and the stratification of country income level of studies. We assessed each of these four components as having minor, moderate, or substantial concerns regarding the specific component. Based on an overall assessment of methodological quality, relevance, adequacy, and coherence, the confidence in the evidence for each review finding was assessed as high, moderate, or low.

### Reporting

This systematic review is reported following the ENTREQ statement guidelines to enhance transparency in reporting qualitative evidence synthesis (S5 Table) [43].

## Results

### General Overview

**Fig 1:**
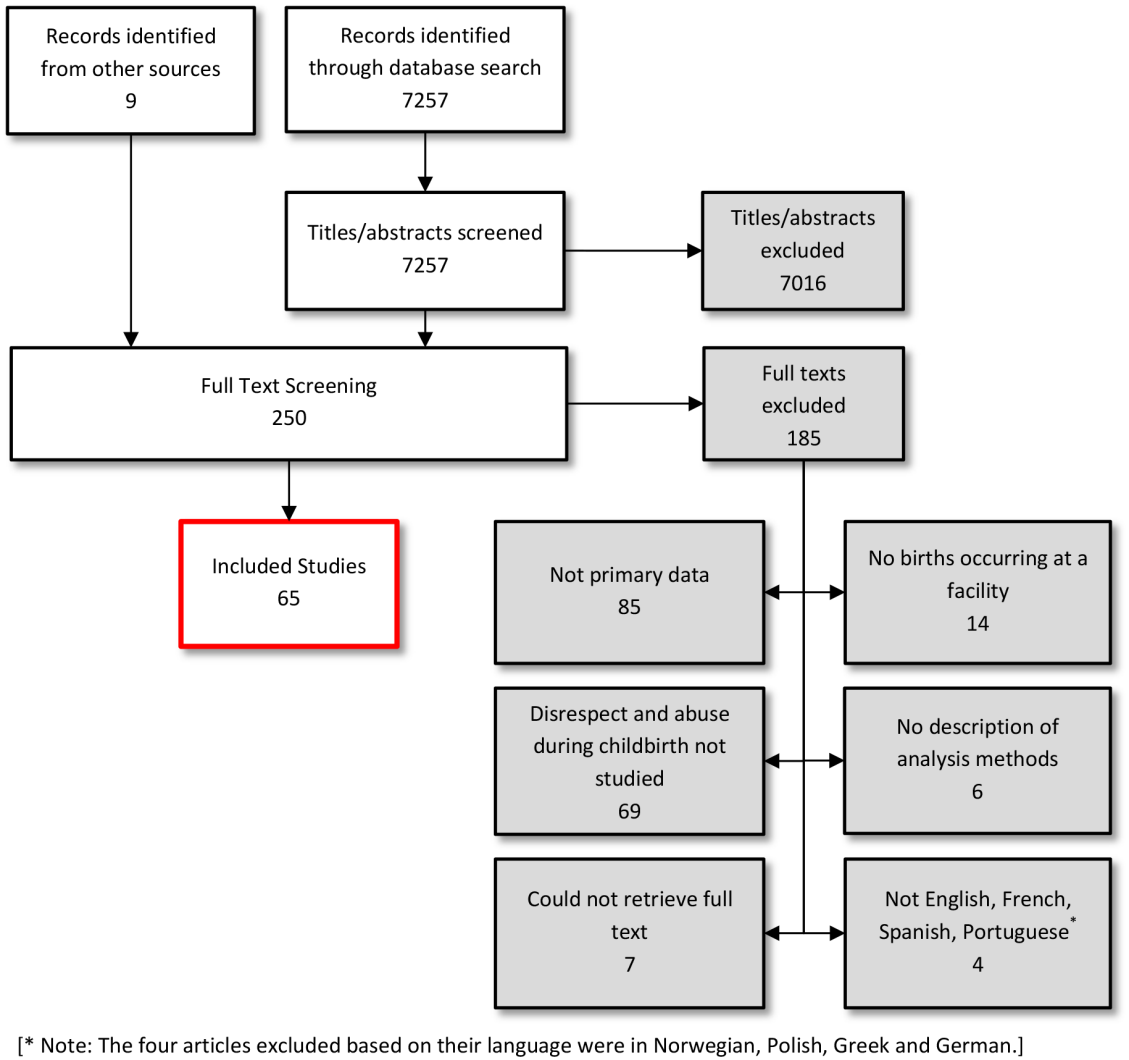
Flow diagram of search and study inclusion process.

The initial PubMed, CINAHL, and Embase searches yielded 5,733 articles, and the updated searches yielded an additional 1,524 articles, for a total of 7,257 articles. Full texts were retrieved for 250 potentially eligible studies. After exclusions, 65 studies were included (Fig 1). The analysis synthesizes findings from primary research conducted across 34 countries: 11 countries in sub-Saharan Africa, five in Asia, two in Oceania, four in Europe, five in the Middle East and North Africa, two in North America, and five in Latin America. Study summaries are presented in S6 Table.

Table 1 presents the typology of the mistreatment of women during childbirth developed from the synthesis of the qualitative and quantitative evidence. First, the qualitative evidence was synthesized into first-order descriptive themes and second- and third-order analytic themes (discussed in detail in the qualitative synthesis below). Then, these themes were compared to the quantitative findings. Most of the quantitative findings fit into the themes constructed from the qualitative synthesis. However, two themes emerged from only the quantitative synthesis, which were then integrated into the typology: sexual abuse and the performance of unconsented surgical operations. These two themes are therefore presented in Tables 1 and 2 but are not discussed in the qualitative synthesis section below.

**Table 1:** Typology of the mistreatment of women during childbirth.

Third-Order Themes	Second-Order Themes	First-Order Themes
**Physical abuse**	Use of force	Women beaten, slapped, kicked, or pinched during delivery
	Physical restraint	Women physically restrained to the bed or gagged during delivery
**Sexual abuse**	Sexual abuse	Sexual abuse or rape
**Verbal abuse**	Harsh language	Harsh or rude language
		Judgmental or accusatory comments
	Threats and blaming	Threats of withholding treatment or poor outcomes
		Blaming for poor outcomes
**Stigma and discrimination**	Discrimination based on sociodemographic characteristics	Discrimination based on ethnicity/race/religion
		Discrimination based on age
		Discrimination based on socioeconomic status
	Discrimination based on medical conditions	Discrimination based on HIV status
**Failure to meet professional standards of care**	Lack of informed consent and confidentiality	Lack of informed consent process
		Breaches of confidentiality
	Physical examinations and procedures	Painful vaginal exams
		Refusal to provide pain relief
		Performance of unconsented surgical operations
	Neglect and abandonment	Neglect, abandonment, or long delays
		Skilled attendant absent at time of delivery
**Poor rapport between women and providers**	Ineffective communication	Poor communication
		Dismissal of women’s concerns
		Language and interpretation issues
		Poor staff attitudes
	Lack of supportive care	Lack of supportive care from health workers
		Denial or lack of birth companions
	Loss of autonomy	Women treated as passive participants during childbirth
		Denial of food, fluids, or mobility
		Lack of respect for women’s preferred birth positions
		Denial of safe traditional practices
		Objectification of women
		Detainment in facilities
**Health system conditions and constraints**	Lack of resources	Physical condition of facilities
		Staffing constraints
		Staffing shortages
		Supply constraints
		Lack of privacy
	Lack of policies	Lack of redress
	Facility culture	Bribery and extortion
		Unclear fee structures
		Unreasonable requests of women by health workers

The typology presented in this table is an evidence-based classification system of how women are mistreated during childbirth in health facilities, based on the findings of the evidence syntheses. The first-order themes are identification criteria describing specific events or instances of mistreatment. The second- and third-order themes further classify these first-order themes into meaningful groups based on common attributes. The third-order themes are ordered from the level of interpersonal relations through the level of the health system.

**Table 2:** Selected measures of how women are mistreated during childbirth in health facilities from three measurement studies.

Type of Mistreatment	Kruk et al. [25]				Sando et al. [27]				Okafor et al. [26]	
	Self-Report at Exit (*n* = 1,779 Women)		Self-Report at Home Follow-Up (*n* = 593 Women)		Self-Report at Discharge 3–6 h Postpartum (*n* = 1,954 Women)		Observation of Labor (*n* = 201 Labors)		Self-Report during Immunization Clinic Visit up to 6 wk Postpartum (*n* = 446 Women)	
	*n*	Percent	*n*	Percent	*n*	Percent	*n*	Percent	*n*	Percent
**Any experience of mistreatment**	343	19.48%	167	28.21%	289	14.79%	—	—	437	98.00%
**Physical abuse**										
Physical abuse	51	2.90%	30	5.08%	89	4.55%	—	—	159	35.70%
Physical abuse (slapping, pinching, etc.)	47	2.68%	30	5.10%	—	—	—	—	—	—
Beaten, slapped, or pinched	34	1.94%	20	3.39%	—	—	—	—	32	7.20%
Restrained or tied down during labor	—	—	—	—	—	—	—	—	77	17.30%
**Sexual abuse**										
Rape	4	0.23%	0	0.00%	—	—	—	—	—	—
Sexually abused by health worker	3	0.17%	2	0.34%	—	—	—	—	9	2.00%
**Verbal abuse**										
Shouting/scolding/called stupid*	153	8.71%	78	13.18%	—	—	—	—	19	4.30%
Threatening or negative comments	93	5.28%	68	11.54%	—	—	—	—	—	—
Threat of withholding treatment	73	4.16%	35	6.01%	—	—	—	—	—	—
**Stigma and discrimination**										
Discrimination based on specific patient attributes	—	—	—	—	—	—	—	—	89	20.00%
**Lack of informed consent and confidentiality**										
Non-consented care	1	0.06%	1	0.17%	5	0.26%	—	—	243	54.50%
Woman not asked for consent for vaginal examination in antenatal ward	—	—	—	—	—	—	164	81.59%	—	—
Shaving of pubic hair without consent	—	—	—	—	—	—	—	—	34	7.60%
Disclosure of HIV status without consent	—	—	—	—	—	—	—	—	8	1.80%
**Physical examinations and procedures**										
Non-consent for tubal ligation, sterilization, or hysterectomy*	1	0.06%	0	0.00%	—	—	—	—	23	5.20%
Episiotomy without consent	—	—	—	—	—	—	—	—	114	25.60%
**Neglect and abandonment**										
Neglect/abandonment*	150	8.53%	92	15.54%	—	—	—	—	130	29.10%
Delivery without attendant	68	3.91%	31	5.31%	—	—	—	—	—	—
**Lack of supportive care**										
Denied companionship by the husband or relatives	—	—	—	—	—	—	—	—	63	14.10%
**Loss of autonomy**										
Detention in the health facility	—	—	—	—	—	—	184	91.54%	98	22.00%
Detention in facility for failure to pay*	—	—	—	—	153	7.83%	—	—	76	17.00%
**Lack of resources**										
Bed in postnatal ward was not clean	—	—	—	—	—	—	126	62.69%	—	—
**Lack of privacy**										
Non-confidential care	77	4.39%	36	6.16%	34	1.74%	—	—	116	26.00%
Lack of physical privacy/provision of care without privacy*	77	4.39%	36	6.16%	37	1.89%	131	65.17%	28	6.30%
**Facility culture**										
Request for bribe or inappropriate demands for payment*	31	1.78%	18	3.07%	3	0.15%	—	—	—	—

This table presents selected measures of how women are mistreated during childbirth in health facilities from three measurement studies conducted in Tanzania and Nigeria [25–27]. These selected measures are reported by study and data collection method and are reorganized according to the domains of the mistreatment of women during childbirth presented in the typology in Table 1. Due to similarities in some terminology across studies, some measures have been aggregated for ease of reporting and interpretation (*); however, it is unclear whether the operationalization of the measure was consistent across studies. Sando et al. [27] stratified findings by HIV status and data collection method; this table presents aggregated measures by data collection method for all women. S1 Table presents all relevant quantitative findings from the 12 included quantitative and mixed-methods studies.

### Quantitative Synthesis

In total, 12 studies had relevant quantitative data [25–27,44–52]. However, only three of these studies explored mistreatment of women during childbirth in health facilities as a primary objective [25–27]. Table 2 presents selected quantitative measures of how women are mistreated during childbirth from these three studies. All three studies operationalized the domains of disrespect and abuse during childbirth from the Bowser and Hill landscape analysis [12]; however, the operationalization of these domains varied substantially by study. We present detailed findings below from these three studies that directly measured the mistreatment of women.

The study by Kruk et al. [25] explored mistreatment among women in rural Tanzania and is based on women’s self-reported experiences during a facility exit survey and during a follow-up survey with a sub-sample of the same women 5 to 10 wk postpartum. The proportion of women who reported experiencing any mistreatment during childbirth was 19.5% in the facility exit survey and 28.2% in the follow-up survey [25]. Common specific experiences included “non-dignified care” (facility exit survey: 12.9%, follow-up survey: 18.9%), “shouting or scolding” (facility exit survey: 8.7%, follow-up survey: 13.8%), “neglect” (facility exit survey: 8.5%, follow-up survey: 15.5%), and “physical abuse” (facility exit survey: 2.9%, follow-up survey: 5.1%) [25].

The study by Sando et al. [27] explored whether women living with HIV were more vulnerable to mistreatment during childbirth in Dar es Salaam, Tanzania, and is based on interviews with women at 3 to 6 h postpartum and direct observations of labor. The proportion of women who reported experiencing any form of mistreatment was 12.2% in HIV-positive women and 15.0% in HIV-negative women: women living with HIV were no more or less likely to report mistreatment (*p* < 0.37) [27]. The direct observations of labor recorded the following types of mistreatment: “partitions did not provide privacy” to women during childbirth (HIV-positive women: 94.4%, HIV-negative women: 91.3%) and “women were not asked for consent during vaginal examination” (HIV-positive women: 100.0%, HIV-negative women: 79.8%); “women’s legs tied” during delivery was rarely noted (HIV-positive women: 0.0%, HIV-negative women: 3.3%) [27].

The study by Okafor et al. [26] explored mistreatment in a teaching hospital in southeastern Nigeria, and is based on interviews conducted with a convenience sample of women accessing newborn services at an immunization clinic. Almost all of the women reported at least one kind of mistreatment during childbirth (98.0%) [26]. Women commonly reported physical abuse during childbirth (35.7%), including being “restrained or tied down during labor” (17.3%) and being “beaten, slapped, or pinched” (7.2%); being “sexually abused by the health worker” was reported by 2.0% of the women.

The other nine studies with relevant quantitative data were indirectly relevant to this review, were limited in scope, and varied in their operational definition of the mistreatment of women [44–52]. However, these studies reported on indicators that can be classified under the domains of mistreatment of women during childbirth, as defined by the qualitative evidence synthesis. For example, a pilot randomized controlled trial in South Africa reported that 84.5% of women were not allowed companions during childbirth and 4.3% of women were slapped or struck [44]. A cross-sectional study from Brazil showed that companions were often not allowed in the labor ward (41.8%) or delivery ward (98.6%) and that 9.0% of women were shouted at or slapped during delivery [50]. S1 Table presents the quantitative measures of mistreatment of women during childbirth from all 12 studies with quantitative data.

### Qualitative Synthesis

The majority of the studies included in this review used qualitative methods only, or a mixed-methods approach where only the qualitative data were relevant [6–11,13,21,27,53–97]. Most studies detailed the experiences of women, but some studies also explored the experiences of health care providers, medical administrators, or policy-makers. Table 3 presents the summary of qualitative findings and confidence assessments. Many themes were homogenous across geographical regions and country income levels; regional and income-level sub-analyses are presented where appropriate. Below we highlight key findings across themes constructed from the qualitative evidence synthesis.

**Table 3:** Summary of qualitative findings.

Review Finding	Contributing Studies	Confidence in the Evidence	Explanation of Confidence in the Evidence Assessment
**Physical abuse**			
**Use of force:** Women across the world reported experiencing physical force by health providers during childbirth. In some cases, women reported specific acts of violence committed against them during childbirth, but women often referred to these experiences in a general sense and alluded to beatings, aggression, physical abuse, a rough touch, and use of extreme force. Pinching, hitting, and slapping, either with an open hand or an instrument, were the most commonly reported specific acts of physical violence.	[6,9,10,13,21,61,67,68,73,75,77,80,84,86,87,91,96,97]	High	18 studies with minor to significant methodological limitations. Thick data from 11 countries across all geographical regions, but predominantly sub-Saharan Africa. High coherence.
**Physical restraint:** Women in Tanzania and Brazil reported physical restraint during childbirth through the use of bed restraints and mouth gags.	[86,97]	Low	2 studies with minor to significant methodological limitations. Limited, thin data from 2 countries (Tanzania and Brazil). Extent of coherence unclear due to limited data.
**Verbal abuse**			
**Harsh or rude language:** Across high-, middle-, and low-income countries, verbal abuse of women by health providers during childbirth was a commonly reported event, particularly the use of harsh or rude language. Women’s perceptions of their facility-based childbirth experiences were often shaped by negative encounters with health workers in which they were verbally abused.	[6,7,9,10,13,48,51–55,58,59,61,63,64,67,68,70,73,75,77,80,81,83,85,87,88,90,91,93]	High	31 studies with minor to significant methodological limitations. Thick data from 18 countries across all geographical regions, but predominantly sub-Saharan Africa. High coherence.
**Judgmental or accusatory comments:** Women reported feeling shamed by health workers who made inappropriate comments to them regarding their sexual activity. Insensitive comments may be experienced more frequently by adolescent or unmarried women, since many communities view pregnancy and childbirth as appropriate only in the context of marital relationships. Intentionally lewd comments humiliated the women while they were in an already vulnerable position during childbirth and in need of supportive care. As a result, women often felt that their health provider was disrespectful, uncaring, and rude.	[10,13,55,58,59,73,77,80,87,91]	Moderate	10 studies with minor to significant methodological limitations. Fairly thick data from 8 countries, predominantly low-income countries. High coherence.
**Threats and blaming:** Some women were threatened with poor quality of care or poor outcomes for their babies as a result of their behavior during childbirth. This included threats of a beating if the woman did not comply with a health worker’s request and threats of withholding health services. Other women were blamed for their baby’s or their own poor health outcomes.	[13,58,59,63,68,77]	Moderate	6 studies with minor to significant methodological limitations. Adequate data from 5 countries, predominantly middle- and high-income countries. High coherence.
**Stigma and discrimination**			
**Discrimination based on ethnicity/race/religion:** Women commonly reported feeling discriminated against due to their ethnic or racial backgrounds. Differential treatment by health workers often pervaded their experiences during childbirth and influenced the quality of care they received. This type of treatment tended to make women feel alienated from their health care providers. In some settings, migrants and refugees received particularly disrespectful care and may have been expected to pay higher rates for services or to pay bribes. This included Somali women with female genital cutting in Canada, Roma women in the Balkans, and refugee women in South Africa.	[8,13,49,52,53,58,62,63,67,72,78,80,95]	High	13 studies with minor to significant methodological limitations. Thick data from 10 countries across all geographical regions and country income levels. High coherence.
**Discrimination based on age:** Women believed that they were discriminated against based on their age, for being pregnant either as an unmarried adolescent or as an older woman of high parity. Adolescents were criticized and ridiculed for engaging in sexual activity before marriage, and some felt that their confidentiality was breached due to their age. Adolescents in South Africa reported that mistreatment that they or their friends experienced during facility-based childbirth directly influenced them to deliver at home in the future.	[9,55,63,67,77,80,88]	Moderate	7 studies with minor to significant methodological limitations. Fairly thick data from 5 countries, but particularly in South Africa. High coherence.
**Discrimination based on socioeconomic status:** Across the world, women who were of lower socioeconomic status reported feeling discriminated against due to their social class or income level. They believed that they received poorer treatment or were neglected because they were poor and often unable to pay for services or to pay bribes. They often felt that health workers humiliated them for their poverty, for their inability to read or write, or for residing in rural or slum areas. Fear of such discrimination was considered a powerful disincentive to deliver in health facilities in Ghana, Sierra Leone, and Tanzania.	[8–10,58,62,67,75,76,79,90,91,93]	High	12 studies with minor to significant methodological limitations. Thick data from 13 countries (1 multi-country study), but predominantly in sub-Saharan Africa. High coherence.
**Discrimination based on medical conditions:** Some women in Kenya and South Africa believed that their positive HIV status contributed to the provision of substandard care, including delays in receiving essential interventions, avoidance of patient contact, and fewer vaginal examinations. However, some health workers in Kenya stated that there was no discrimination against or segregation of HIV-positive women in the labor ward, although they reported being “anxious” if they suspected a woman was HIV-positive and might have handled such women with “extra care.”	[11,13,27]	Low	3 studies with minor to significant methodological limitations. Adequate data from 3 countries (South Africa, Kenya, and Tanzania). Reasonable level of coherence; the finding may have higher confidence in settings with similar HIV epidemics or where there may be discrimination based on other medical conditions.
**Failure to meet professional standards of care**			
**Painful vaginal exams:** Some women reported frequent and painful vaginal examinations during labor. They viewed the number of vaginal examinations they received during labor as excessive and dehumanizing. In some cases, vaginal examinations were conducted in non-private settings and women may not have consented to the procedure, or the procedure may not have been communicated to them.	[54,58,74,80,83,86,89,95]	Moderate	8 studies with minor to significant methodological limitations. Fairly thick data from 5 countries across multiple geographical regions and country income levels. High coherence.
**Refusal to provide pain relief:** Across multiple settings, women described health workers’ refusal to provide pain relief or pain medication not being available for them during labor. Surgical procedures, such as episiotomy, were sometimes carried out without any pain relief. In lower-resource settings, this was often due to stock outs or lack of sufficient patient payment. In higher-resource settings, women reported that they were not offered pain relief or were denied pain relief requested.	[13,21,58,68,75,77,80,81,90,92,93]	High	11 studies with minor to moderate methodological limitations. Thick data from 9 countries across multiple geographical regions and country income levels. High coherence.
**Lack of informed consent process:** Women complained that they were not always asked to provide consent for medical procedures such as cesarean section. When women were asked to provide consent prior to a procedure, they were not always adequately informed of the risks and benefits of the procedure and felt that the health worker went through the motions of obtaining consent. Some women in Kenya also avoided or feared facility-based delivery due to anxiety about being tested for HIV without their consent.	[11,13,92]	Moderate	3 studies with minor to moderate methodological limitations. Fairly thick data from 3 countries (Kenya, South Africa, and United Kingdom). High coherence.
**Breaches of confidentiality:** Some women complained that the health workers did not maintain doctor–patient confidentiality and disclosed private information either to their male partners or to other patients. For some HIV-positive women in Kenya, the lack of trust in the confidentiality of treatment at health facilities was so great that they chose to deliver at home, where their HIV status would not be disclosed to other community members or health workers.	[11,13,27,55,59]	Moderate	5 studies with minor to significant methodological limitations. Fairly thick data from 5 countries, particularly in sub-Saharan Africa. High coherence.
**Neglect**, **abandonment**, **or long delays:** Women frequently referred to long delays in receiving care and inattentive health workers who neglected women during labor and delivery. Women commonly reported feeling alone, ignored, or abandoned during their stay at the facility, and felt as if their request for help or attention from health workers was an imposition. Many women reported long wait times before seeing a health worker or before receiving an intervention. Long wait times may have been exacerbated when women did not book prior to delivery, as their information may not have been in the system, and they perceived that health workers punished women who did not book ahead with longer wait times. These experiences of neglect and abandonment by health workers in facilities were direct barriers to seeking future deliveries in facilities in Ghana, Bolivia, and Tanzania, as some women prioritized the need for supportive childbirth care, which they could receive from traditional providers.	[6–10,13,21,48,51,59,62–64,66–68,70,71,75–78,80,81,84,86–88,92–95,97]	High	33 studies with minor to significant methodological limitations. Thick data from 21 countries across all geographical and country income levels. High coherence.
**Skilled attendant absent at time of delivery:** Some women reported that health worker shortages and negligence directly increased the physical risks women faced during delivery. In some extreme cases of neglect, women delivered at facilities without the presence of skilled birth attendants, who were preoccupied with other tasks.	[9,13,21,48,59,67,77,81,84,86,87,93]	High	12 studies with minor to significant methodological limitations. Thick data from 8 countries, particularly in the Middle East and sub-Saharan Africa. High coherence.
**Poor rapport between women and providers**			
**Poor communication:** Women commonly referred to communication issues between health workers and themselves that left women feeling “in the dark” about their childbirth care. Many women felt dissatisfied with the information and explanations provided to them by health workers regarding their care and believed that the health workers were more interested in having them comply with their demands than in allowing the women to ask questions to clarify the proposed procedures. These experiences made women feel distanced from health workers, fearful of procedures, and like they were not active participants in their childbirth experience. Some women in the United Kingdom, Dominican Republic, and Brazil reported believing that health workers intentionally avoided exchanging information with patients and described health workers as unresponsive to patient needs.	[6,8,11,13,21,48,50,52,53,57,58,60,62,64,66,67,70,73,75,78,84,86,88,92–94,96,97]	High	28 studies with minor to significant methodological limitations. Thick data from 22 countries across all geographical regions. High coherence.
**Language and interpretation issues:** Women often suffered from language and interpretation barriers when attempting to communicate with health workers, and this was particularly a burden for migrant and refugee women in high-income settings.	[8,13,52,58,62,78]	Moderate	6 studies with minor to moderate methodological limitations. Fairly thick data from 6 middle- and high-income countries. High coherence.
**Lack of supportive care from health workers:** Women commonly reported a lack of supportive care during childbirth in facilities, including the perception that the care provided by health workers was mechanical and lacked comfort and courtesy. During their deliveries, women often felt that they did not receive the time and attention from health workers to make them feel supported and adequately cared for. Women felt that staff were insensitive to their needs, which made women feel unconfident, anxious, and alone. Many women believed that delivering in a health facility would ensure positive health outcomes for themselves and their babies. However, while they often felt that they received technically sound care, their experiences at the facility were marred by feelings of being emotionally unsupported. Women felt that they were provided with systemized, mechanistic care that focused solely on technical outcomes rather than supportive care that incorporated sensitive communication and a comforting touch. Women from Sierra Leone, Uganda, and rural China stated that when expectations of a supportive environment during a facility-based childbirth were not met, they may be less inclined to deliver at a facility in future births.	[6,7,9,21,48–50,52,57,58,60,61,63,65,66,71–73,75,78,81,82,88,90,92,93]	High	26 studies with minor to significant methodological limitations. Thick data from 21 countries across all geographical regions, but predominantly in sub-Saharan Africa. High coherence, but lack of supportive care in lower-income settings may impact future childbirth care-seeking behaviors.
**Denial or lack of birth companions:** Women desired the supportive attention and presence of a birth companion, who may be a family member, husband, or a friend. However, women across the world were often prohibited from having a companion of their choice during delivery. Although not always clearly explained to clients, it was often official hospital policy to ban birth companions, as they were deemed unnecessary by the administration. The lack of companionship left women feeling disempowered, frightened, and alone during childbirth as they yearned for the comfort provided by familiar faces.	[6,9,21,48–50,54,66,72,75,78,90]	Moderate	12 studies with minor to significant methodological limitations. Fairly thick data from 9 countries across many regions, but predominantly middle-income settings. High coherence.
**Lack of respect for women’s preferred birth positions:** Some women preferred to deliver in positions other than the supine position, such as by squatting or kneeling, and resented that health workers forced them to deliver in undesirable or humiliating positions. Women felt that adopting an undesirable birth position at the demand of the health worker made them passive participants in their childbirth process. Restricting the childbirth position to lying down acted as a barrier for some women to access facility-based deliveries in Bangladesh. Health workers in Bangladesh, Cuba, and Uganda explained that they had not been trained to deliver women in positions other than lying down and felt uncomfortable letting a woman choose her own birth position.	[6,9,21,53,70,72,82,89]	Moderate	8 studies with minor to significant methodological limitations. Adequate data from 7 countries, predominantly middle-income countries. Reasonable level of coherence.
**Denial of safe traditional practices:** Some women in Ghana and the United Kingdom referred to the denial of safe traditional religious or cultural practices related to childbirth. Maintaining these traditional practices, such as retaining the placenta for burial, were important to women, and the denial of these practices may be an important barrier to seeking facility-based delivery or experiencing quality supportive care.	[10,78]	Low	2 studies with minor to moderate methodological limitations. Fairly thin data from 2 counties (United Kingdom and Ghana). Extent of coherence unclear due to limited data, but findings were similar across the studies.
**Objectification of women:** In several settings, women reported feeling stripped of their dignity during childbirth due to the health workers’ objectification of their bodies. They resented being forced to be on all fours and exposing their bodies to numerous health workers, sometimes including large groups of students.	[13,21,48,57,84]	Moderate	5 studies with minor to significant methodological limitations. Adequate data from 8 countries (1 multi-country study), but only in middle- and high-income settings. Reasonable level of coherence for middle- and high-income settings.
**Detainment in facilities:** Studies from Benin and Sierra Leone suggest that either the mother or baby may be detained in the health facility, unable to leave until they pay the hospital bills.	[73,90]	Low	2 studies with moderate methodological limitations. Fairly thin data from 2 countries (Benin and Sierra Leone). Extent of coherence unclear due to limited data, but findings were similar across the studies.
**Health systems conditions and constraints**			
**Physical condition of facilities:** Both women and health workers described the physical conditions of health facilities that contributed to the mistreatment of women. Antenatal and delivery wards were described as “dirty,” “noisy,” “disorderly,” or “overcrowded,” or with needles, biomedical waste, or dirt strewn on the floor.	[27,53,59,67,70,84,95,96]	Moderate	8 studies with minor to significant methodological limitations. Fairly thick data from 8 low- and middle-income countries. High coherence.
**Staffing shortages:** Both women and health workers illustrated how staffing shortages affected the quality of care provided. Staffing shortages were of particular concern in low- and middle-income countries and often led to longer wait times for women and their families, as well as neglectful or poor-quality care. Women and health workers both purported that staffing shortages not only affected direct provision of care but also contributed to the health workers’ negative attitudes or lack of motivation. In low- and middle-income countries, providers of all cadres were described as “overworked,” “too busy,” “stretched,” and “underpaid” by both women and other providers.	[13,51,78,84,87,90,91,93]	Moderate	8 studies with minor to significant methodological limitations. Fairly thick data from 7 countries, particularly in sub-Saharan Africa. High coherence.
**Staffing constraints:** In addition to the understaffing of health workers, inexperienced or poorly trained health workers were often responsible for inappropriate levels of care without supportive supervision. In lower-level facilities, qualified physicians may be a rarity, leaving unskilled nurses to attend to labor management, complications, and decisions regarding referrals.	[53,54,84,86, 87,96]	Low	6 studies with minor to significant methodological limitations. Adequate data from 6 countries. High coherence.
**Supply constraints:** Health workers and male partners explained that there were often inadequate medical supplies, including medication, gloves, and blood, which are critical for health workers to execute their duties. In some cases, this shortage led to the requirement that patients bring their own supplies, such as gloves, gauze, and pads. This may have caused health workers to attend first to women who brought their own supplies, or for women to think that the health workers were withholding supplies from them for malicious reasons. Health workers believed that the shortage of supplies, particularly gloves, caused unnecessary danger and stress in the work environment.	[9,27,54,61,67,70,87,93,96]	Moderate	9 studies with minor to significant methodological limitations. Thick data from 7 low- and middle-income countries. High coherence for low- and middle-income settings.
**Lack of privacy:** Women across many settings reported a general lack of privacy in the antenatal and labor wards and specifically during vaginal and abdominal exams. Women were exposed to other patients, their families, and health workers due to the lack of curtains to separate them from other patients, the lack of curtains on the outside windows, and doors that were left open. In low- and middle-income countries, the antenatal and labor/delivery wards were sometimes common or public areas, and women were sometimes forced to share beds with other parturient women who may be strangers. Not surprisingly, women expressed their desire to be shielded from other patients, male visitors, and staff who were not attending them while they were in labor and particularly during physical exams. They felt that such exposure, particularly during this vulnerable time, was undignified, inhumane, and shameful.	[11,21,49,53,54,58,70,74,75,84,95,96]	High	12 studies with minor to significant methodological limitations. Thick data from 11 countries across all geographical and income-level settings. High coherence.
**Lack of redress:** Women lamented the inability to express their opinions about the treatment and services rendered during childbirth. Several reasons for this were posited, including women fearing unfair treatment or discrimination if they complained, women being unaware of their rights as patients, fear of facility closure, and a lack of a redress or accountability mechanism for lodging complaints. Even in settings where health policies dictated the creation of a formal complaint registration system, these systems may not have been implemented at a facility level. The lack of accountability and sanctioning within the health system left women feeling vulnerable and powerless to seek justice for their mistreatment.	[8,9,13,67,77]	Moderate	5 studies with minor to significant methodological limitations. Adequate data from 4 countries (1 multi-country study), but 3 studies are from South Africa. Reasonable level of coherence.
**Bribery and extortion:** In several settings, women reported the need to pay bribes to different workers throughout health facilities, including to doctors, nurses, midwives, receptionists, and guards. Bribes took the form of money, food, drinks, or other gifts. Women believed that paying bribes positively influenced the quality of services provided to them in health facilities. For instance, bribery could ensure that women received timely care, adequate attention from health providers, and any necessary drugs or medications. Health workers were perceived to ignore women in the maternity ward until the patients paid the bribe, at which point, the health workers would become attentive to their needs. One study from the Balkans explicitly stated that Roma women avoided facility-based deliveries because they know that bribes are required to receive sufficient care.	[8,9,13,56,71,75,76,93]	Moderate	8 studies with minor to significant methodological limitations. Fairly thick data from 8 countries, but predominantly in sub-Saharan Africa. Reasonable level of coherence.
**Unclear fee structures:** Women in Tanzania reported that an unclear fee structure for services and supplies rendered during childbirth led to frustration, confusion, and a fear of detainment in the facility.	[9]	Low	1 study with minor methodological limitations. Fairly thick data, but only from Tanzania. Coherence could not be assessed as only 1 contributing study.
**Unreasonable requests of women by health workers:** In South Africa and Ghana, women were angry at health workers for making unreasonable demands of them during their stay at health facilities. In particular, women were forced to clean up the “mess” they made on the floor or bed immediately after both vaginal deliveries and cesarean sections, when women were feeling particularly weak and vulnerable. Some women were told to walk to a different room, to retrieve supplies or to dispose of medical waste during the second stage of labor or immediately after delivery, without a wheelchair or support from birth attendants.	[6,7,13,67,77]	Moderate	5 studies with minor to significant methodological limitations. Fairly thick data from 2 countries (South Africa and Ghana). High coherence.
**Impact on utilization of maternal health services**			
**Power dynamics and systemized abuse:** Health workers discussed how the hierarchical authority in the health system legitimized the control that health workers have over their patients and contributed to the detrimental treatment of women during childbirth. These power differentials place women at the bottom of the hierarchy, where their needs and concerns were often ignored or deemed as unimportant by health workers. Furthermore, the lack of supportive supervision for health workers from their superiors contributed to feelings of demoralization and negative attitudes, thus perpetuating the mistreatment of women. As a result of past negative experiences, both health workers and patients may have come to expect and accept the poor treatment of women as the norm.	[10,13,59,77,91]	Moderate	5 studies with minor to significant methodological limitations. Fairly thick data from 4 low- and middle-income countries. High coherence.
**Impact on future care-seeking behaviors**, **late attendance to facilities**, **and desire for home birth:** Experiences of mistreatment during childbirth may have far reaching consequences for women and communities outside of the direct patient–provider interaction. Prior experiences and perceptions of mistreatment, low expectations of care provided at facilities, and poor reputations of facilities in the community eroded many women’s trust in the health system and may impact their decision to deliver in a health facility in the future, particularly in low- and middle-income countries.	[6,8–10,13,21,52,53,61,64,71,77–79,82,85,90,94,96]	High	19 studies with minor to significant methodological limitations. Thick data from 16 countries, but particularly in low- and middle-income countries and sub-Saharan Africa. High coherence.

A summary of the review findings from the qualitative synthesis are presented here, with the relevant studies contributing to each review finding. The confidence in the evidence refers to the overall CERQual assessment of the methodological limitations of included studies, relevance, adequacy, and coherence, and is rated as high, moderate, or low. The explanation of the assessment of the confidence in the evidence provides a brief assessment of each CERQual domain to support the overall CERQual assessment.

### Physical Abuse

Physical abuse during childbirth [9,10,13,21,61,67,68,73,75,77,80,84,86,87,91,97] was perpetrated by nurses [10,13,67,80,84,86], midwives [61,73,75,77,87,91], and doctors [84,91]. Women sometimes reported specific acts of violence, but often referred to these experiences more generally, describing beatings, aggression, physical abuse, a “rough touch,” and the use of extreme force [9,10,13,21,61,73,80,84,87]. Hitting and slapping, with an open hand or an instrument, were the most commonly reported specific acts of physical violence [10,13,67,75,77,87,91]. Women also reported being pinched, particularly on the thighs [13,86] and kicked [10]. Some women were physically restrained during labor with bed restraints [97] and mouth gags [86].

### Verbal Abuse

Verbal abuse of women by health providers during childbirth was commonly reported across all regions and country income levels [6,7,9,10,13,48,51–55,58,59,61,63,64,67,68,70,73,75,77,80,81,83,85,87,88,90,91,93]. Verbal abuse included the use of harsh or rude language [6,7,9,10,13,48,51–55,58,59,63,64,67,73,75,77,80,81,83,85,87,88,90,93], judgmental or accusatory comments [9,13,54,55,58,59,63,68,73,77,80,87], and threats of poor outcomes or withholding treatment [59,63,75,77,91]. Where the cadre of health worker perpetrating the verbal abuse was specified, nurses and midwives (with whom women have the most contact) were most commonly mentioned [6,7,9,10,13,48,51–53,58,59,63,67,68,70,73,75,77,80,81,83,87,90,93], followed by doctors [58,59,81] and administrative staff [52,67,77]. Women who were from a lower socioeconomic status, were migrants, or were from an ethnic minority were sometimes called derogatory slurs during delivery [13,52,53,58,77,80]. Women felt shamed by health workers who made inappropriate comments to them regarding their sexual activity [10,13,54,55,58,59,73,77,80,87,91], particularly adolescent or unmarried women [55,77,80]. Health workers also ridiculed and admonished women for certain behaviors such as their inability to breastfeed, their failure to attend antenatal care, and the absence of their partner during childbirth [13,58,63,68,77]. In Canada, refugee women who had experienced female genital cutting reported judgmental remarks from their health providers regarding the appearance of their genitalia [58]. Some women were threatened with poor quality of care, withholding of treatment, or poor outcomes for their babies as a result of their behavior during childbirth [13,58,59,63,68,77], including threats of beatings if the woman was noncompliant [77], threats of withholding services [59], and blame for their baby’s or their own poor health outcomes [75,77].

Women’s childbirth experiences were negatively impacted by these abusive encounters with health workers. Box 1 provides a list of the many words women used to describe the types of verbal abuse perpetrated by health workers. Some women believed that their treatment by health workers was contingent on their ability or inability to remain silent throughout labor and delivery [53,80], or that they were poorly treated because of their disobedience in the antenatal or delivery ward, such in as pushing before instructed to do so [7,9,10,53,63,77,80,81].

**Box 1. Words Women Used to Describe Types of Verbal Abuse from Health Workers**

- “rude” [6,13,52,53,58,63,67,75,77,80,85,97]
- “harsh language” [10,77,80]
- “sarcasm” [77]
- “swear” [63]
- “snap at” [77]
- “mock” [13,51,73]
- “threaten” [13,55,63,77]
- “scold” [9,59,67,77,80,81]
- “scream”/“shout”/“yell” [7,9,10,13,48,51,52,59,63,64,75,77,80,81,85,91]
- “degrade”/“belittle”/“dehumanize” [48,59,81]
- “intimidate” [63,70]
- “raise voice” [80]
- “ridicule” [77,80]
- “name-calling” [13,52,53,58,80]
- “humiliate” [6,58,73,77,80,85]
- “insult” [10,13,51,67]

### Stigma and Discrimination

Stigma and discrimination during facility-based childbirth occurred across four main categories: (1) ethnicity/race/religion, (2) age, (3) socioeconomic status, and (4) medical conditions. Women commonly reported feeling discriminated against due to their ethnic or racial background [8,13,49,52,53,58,62,63,67,72,78,80,95]. Differential treatment by health workers influenced the quality of care they received and alienated them from their providers [13,58,63,78]. Women felt that some biomedical models of maternity care disrespected cultural preferences and propagated racial stereotyping [8,63,65,67,78].

Both unmarried adolescents [55,63,67,77,80] and older women of high parity [9,67,88] reported discrimination. Adolescents were criticized and ridiculed for engaging in sexual activity before marriage [55,77,80], and some felt that their confidentiality was breached due to their age [55].

Women of lower socioeconomic status believed that they received poorer treatment because they were unable to pay for services or to pay bribes [8–10,58,62,67,75,76,79,90,91,93]. They felt health workers humiliated them for their poverty, for their inability to read or write, for residing in rural or slum areas, or for being “dirty” or unkempt [8–10,76,79]. Fear of such discrimination was considered a powerful disincentive to deliver in health facilities in Ghana, Sierra Leone, and Tanzania [10,79,90]. Health workers confirmed that women of lower socioeconomic status were more likely to receive poorer treatment [91].

Women with HIV believed that their positive HIV status contributed to the provision of substandard care, including delays in essential interventions, avoidance of patient contact, and fewer vaginal examinations [11,13,27]. However, some health workers in Kenya stated that there was no discrimination against or segregation of HIV-positive women in the labor ward, although they reported being “anxious” if they suspected a woman was HIV-positive and might have handled such women with “extra care” [11,27].

### Failure to Meet Professional Standards of Care

Health workers often failed to meet professional standards of care intended to address the basic needs of women during childbirth, particularly regarding (1) lack of informed consent and confidentiality, (2) improper conduct of physical examinations and medical procedures, and (3) neglect and abandonment of women.

#### Lack of informed consent and confidentiality

Women complained that they did not provide consent for medical procedures such as cesarean section [13,92]. When women were asked to provide consent prior to a procedure, they were not always adequately informed of the risks and benefits and felt that the health worker only went through the motions of obtaining consent [13]. Some women in South Africa avoided or feared facility-based delivery due to anxiety about HIV tests given without consent [11].

Women complained that health workers did not maintain doctor–patient confidentiality and disclosed private information to male partners or other patients [11,13,27,55,59]. For some HIV-positive women, the lack of trust in the confidentiality of treatment at health facilities was so great [27] that they chose to deliver at home, where their HIV status would not be disclosed to other community members or health workers [11].

#### Physical examinations and procedures

Many women reported frequent and painful vaginal examinations during labor [58,74,80,83,86,89,95], which they viewed as excessive [58,74,83,86] and dehumanizing [58,74,83,89]. Vaginal examinations were sometimes conducted in a non-private setting [74], and women may not have consented to examinations [74,89].

Health workers sometimes withheld pain relief, or pain medication was not available for women during labor, often due to stock outs or insufficient patient payment [13,21,58,68,75,77,80,81,90,92,93].

#### Neglect and abandonment

Women frequently referred to long delays in receiving care and inattentive health workers who neglected women during labor and delivery [6,8–10,13,21,48,51,59,62–64,66–68,70,71,75–78,80,81,84,86–88,92–95,97]. Women reported feeling alone, ignored, and abandoned during their stay at the facility, and felt that their request for help or attention from health workers was an imposition [6,8–10,48,63,64,66,71,75–78,80,81,84,86–88,92,93,97]. Interactions with health workers were “rushed,” and women felt like a “burden” or a “nuisance” or that they were “bothering” the health workers or “putting them out” [77,78,92]. As such, women did not feel that consideration for their well-being was a central component of their care. Many women reported long wait times before seeing a health worker or receiving an intervention [10,13,77,81,86,87,92,94]. Long wait times were exacerbated when women did not book prior to delivery, because their antenatal care information was not in the system, and some health workers “punished” women with longer wait times if they did not book ahead [77]. Other women waited for many hours or days before receiving referrals to higher-level health facilities [13,86]. In some cases, women who were in labor were refused care at a facility without an exam [13,51,77,81]. Turning women away from health facilities during labor is particularly troublesome for those who live far away from the facility or cannot afford transportation costs [13,51].

Some women reported that neglect directly increased the physical risks women faced during delivery [13,21,48,67,77,81,86,87]. In some extreme cases of neglect, women delivered at facilities without the presence of skilled birth attendants, who were preoccupied with other tasks [6,9,13,21,48,59,67,77,81,84,86,87,93]. In Tanzania, several women reported that they sent escorts to find traditional birth attendants to assist in the delivery in a facility because of neglect by the facility-based providers [9].

### Poor Rapport between Women and Providers

Women commonly described communication issues with health workers that left women feeling “in the dark” about their care [8,11,13,21,48,50,52,53,57,58,60,62,64,66,67,70,73,75,78,84,86,88,92,94,97]. Women often made general statements about poor staff attitudes, without detailing specific interactions [10,13,48,53,63,64,66,75,77,78,86,87,91,93]. Some referred to providers as disrespectful [66], unwelcoming [63], misbehaving [91], having negative attitudes [10,13,48,64,78,87], unsupportive [87,93], judgmental [10], unfriendly [10,75,91,93], unhelpful [53], rude [75], impolite [75], sarcastic [77], discouraging [63], unprofessional [91], or unkind [91].

#### Ineffective communication

Women were often dissatisfied with explanations from health workers regarding their care and believed health workers were more interested in their compliance than in answering questions or clarifying proposed procedures [6,13,21,48,53,61,62,64,73,86,88,92–94]. Health workers actively dismissed women’s concerns and anxieties regarding potential complications or impending delivery [6,21,48,60,62,73,78,88,92,94]. When faced with labor complications, women believed that adequate explanations from health workers were imperative to fully comprehend the situation, but these explanations were often rushed, if provided at all [6,13,57,61,64,67,73,86,88,92–94].

In some cases, the risks of procedures were not properly communicated to women, which in some cases increased women’s fear of the procedure, such as cesarean section [13,61,64,73,92–94]. Women who refused cesarean section felt that the rationale for the surgery was not adequately described [64,73,94] and that where communication between providers and women did take place, it was inadequate [84]. Women also reported that they were not “heard” or respected by their providers [21,48,53,57,60,64,66,92,94].

Women often reported language barriers and interpretation challenges when attempting to communicate with health workers [8,13,52,58,62,78], and this was particularly an issue for migrant and refugee women in high-income settings [52,58,62,78]. In some cases, women were given medication or procedures without knowing their purpose [13,52]. Sometimes, if interpreters were unavailable, family members or other patients were used as interpreters, which made the women uncomfortable [62]. Some women tried to bring companions as interpreters, but their companions were unable to gain access to the antenatal and labor wards [13,62].

#### Lack of supportive care

Women commonly reported a lack of supportive care, including a perception that the care provided by health workers was mechanical or lacked comfort or courtesy [6,7,21,48–50,52,57,58,60,63,65,66,71–73,75,78,81,82,90,92,93,95]. Women often felt that they did not receive the time and attention from health workers to feel supported and adequately cared for [7,21,48,49,52,57,58,60,63,65,71,73,78,81,92,93], which made women feel anxious and alone [21,52,57,58,60,63,92]. Many women believed that delivering in a health facility would ensure positive health outcomes for themselves and their babies. Despite receiving technically sound care, their experiences were marred by a lack of emotional support, and their care experiences were therefore incongruous with their expectations.

While women often desired the presence of a birth companion, such as a family member, husband, or friend [6,9,21,48–50,54,66,72,75,78,90], many were prohibited from having their companion of choice with them during delivery [21,48–50,54,66,72,75]. Although not always clearly explained, hospital policy often banned birth companions, as the administration deemed them an unnecessary hindrance [48–50,54,75]. The lack of companionship left women feeling disempowered, frightened, or alone during their delivery.

#### Loss of autonomy

Women commonly reported feeling a loss of autonomy, including objectification and disrespect of safe traditional practices and birthing positions, which rendered them passive participants [6,7,10,13,21,48,50,53,57,60,63,65–68,70,72,73,79,82,84,89,97]. Women overwhelmingly felt “removed” from decisions about their childbirth, and that health workers were coercive and rushed through their deliveries in an attempt to reduce them to dependent, disempowered, and passive patients [6,48,50,57,60,63,66,67,82,97]. Women reported feeling stripped of their dignity during childbirth due to the health workers’ objectification of their bodies [13,21,48,57,84]. They resented being forced to be on all fours or to expose their bodies to numerous health workers [21,57,84]. Women felt that they were “processed technically” and did not receive humanized or compassionate care [21,48,57]. After delivery, some women were left alone in their own blood, urine, and feces with no support from the health workers to clean up [84]. Women were sometimes denied food and water during labor [48] or were confined to recumbent positions (lying down) rather than upright positions (walking, standing) [21].

Some women preferred to deliver in a squatting or kneeling position, rather than the supine position, and resented feeling forced to deliver in undesirable or humiliating positions that rendered them passive [6,9,21,53,70,72,82,89]. Some health workers explained that they had not been trained to deliver women in positions other than lying down and felt uncomfortable letting a woman choose her own birth position [53,72,82].

Maintaining safe traditional practices, such as retaining the placenta for burial, were important to women, and the denial of these practices may be an important barrier to seeking facility-based delivery or experiencing quality supportive care [10,78].

Select studies from Benin, Tanzania, and Sierra Leone suggest that either the mother or baby may be detained in the health facility, unable to leave until they pay the hospital bills [9,73,90]. However, this phenomenon was not richly described in the primary studies.

### Health System Conditions and Constraints

While women and providers most often discussed individual-level experiences and factors contributing to the mistreatment of women during childbirth in health facilities, they also discussed greater health system factors that contributed to an abusive environment and culture within a facility [10,11,13,21,49,51,53,58,59,67,74,75,77,78,84,87,90,91]. Most women and providers who discussed health system factors believed that health workers were doing the best that they could in constrained environments, particularly in low- and middle-income countries.

#### Lack of resources

Both women and health workers illustrated how staffing constraints affected the provision of care [13,51,54,61,70,78,84,87,90,91,93]. Staffing shortages were of particular concern and led to longer wait times and neglectful and poor-quality care [13,51,61,70,78,84,87,90,91]. Providers of all cadres were described as “overworked,” “too busy,” “stretched,” and “underpaid” by both women and other providers [13,51,78,87,91,93]. Where health facilities were understaffed, women were triaged, which may contribute to women who are experiencing normal labor feeling neglected [70,84,87]. Furthermore, inexperienced or poorly trained health workers were often responsible for inappropriate levels of care, without adequate supervision [53,84,87]. In lower-level health facilities, qualified doctors may be rare, leaving unskilled nurses or inexperienced medical officers to attend to labor management, complications, and decisions regarding interventions and referral [53,84,87]. Staffing constraints are also a critical factor in experiences of neglect, as there are often not enough health workers available to engage with women. Women and health workers both suggested that staffing constraints not only directly affected provision of care, but also contributed to the health workers’ negative attitudes and poor motivation [78,87,91].

Women and health workers also reported a lack of privacy in the antenatal and labor wards, particularly during vaginal and abdominal exams, with problems including no curtains to separate women from other patients [11,21,49,53,54,58,61,70,74,75,84,95]. In low- and middle-income settings, the antenatal and labor/delivery wards were sometimes located in common or public areas, and some women were forced to share beds with other parturient women [11,21,49,70]. Women expressed their desire to be shielded from other patients, male visitors, and staff who were not attending them while they were in labor and, particularly, during physical exams [11,21,49,53,58,70,74,75,84]. They felt that such exposure was undignified, inhumane, or shameful [21,49,53,74,75].

Health workers reported having inadequate medical supplies, such as medication, gloves, and blood, which caused unnecessary danger and stress in their work environment [9,27,54,61,67,70,87,93].

Both women and health workers described antenatal and delivery wards that were “dirty,” “noisy,” “disorderly,” or “overcrowded,” or with needles, biomedical waste, or dirt strewn on the floor [6,27,53,59,61,67,70,84,95]. In some facilities, women in labor lay on bare mattresses that were soiled with urine, feces, blood, vomit, and amniotic fluid [6,84].

#### Lack of policies

Women lamented their inability to express their opinions about the treatment and services rendered during childbirth [8,9,13,67,77]. Several reasons for this were posited, including women not wanting to get a health worker in trouble [67], women fearing unfair treatment or discrimination if they complained [9,13,67], health workers being perceived as unapproachable [67], women being unaware of their rights as patients [8,13,67], fear of facility closure [9], and a lack of a redress or accountability mechanism for lodging complaints [8,9,13,67]. Even in settings where health policies dictated the creation of a formal complaint registration system, these systems were not always implemented [13]. When complaints were launched through informal mechanisms, facility-level responses were seldom received, thus discouraging future complaints [13]. The lack of accountability and sanctioning within the health system left women feeling powerless to seek justice for their mistreatment.

#### Facility culture

Women reported the need to pay bribes in health facilities [8,9,13,56,71,75,76,93], including to doctors [8,71], nurses [8,13,56], midwives [13,75], receptionists [13], and guards [13]. Bribes took the form of money [8,13,56,71,75], food or drinks [13,56], jewelry [8], or other gifts [8,13]. Women believed that paying bribes could ensure the receipt of timely care, adequate attention from health providers, and medication [8,9,13,56,71,76,93]. Health workers were perceived to ignore women in the maternity ward until they paid a bribe [8,13,56,71,76,93]. Women in Tanzania reported that an unclear fee structure for services and supplies rendered during childbirth led to frustration, confusion, and a fear of detainment in the facility [9].

Some women were angry with health workers for making unreasonable demands of them, such as being forced to clean up the “mess” they made on the floor or bed after vaginal deliveries and cesarean sections [6,7,13,67,77]. Some women were told to walk to a different room to retrieve supplies or to dispose of medical waste during advanced labor or immediately after delivery, without a wheelchair or support [7,13,67,77].

### Exploring the Influences of the Mistreatment of Women

#### Potential drivers of the mistreatment of women

Both women and health workers posited potential drivers for the mistreatment of women during facility-based childbirth [10,13,21,59,67,72,73,75,77,80,84,87,91,93]. Women referred to health workers as impatient and hurried [21,73,75,84,91], and stated that they used force to gain compliance [10,13,21,73,77,87]. Some women believed that health workers sought to ensure good health outcomes, and women were therefore yelled at because of their disobedience in the antenatal or delivery ward, such as in pushing before instructed to do so, or because of the health workers being “overstretched,” “tired,” or “overworked” [7,9,10,53,63,67,77,80,81,93].

Health workers described how hierarchical authority in the health system legitimized the control health workers had over women during childbirth [10,13,54,59,77,91]. Some providers believed that they could use extreme or coercive measures to gain compliance from women [77], and some providers did not feel obligated to provide care when women were noncompliant [77]. Furthermore, the lack of provider supervision contributed to feelings of demoralization and negative attitudes [13], leading to both health workers and patients expecting and accepting the poor treatment of women as the norm [13,91].

Some health workers blamed health system issues—such as understaffing, high patient volume, low salaries, long hours, and lack of infrastructure—for creating stressful environments that facilitated unprofessional behavior [13,70,91,93]. Some midwives felt that poor health outcomes were inevitable in their work environment because women arrived at the facilities with complications, and that they were unfairly blamed for mistreatment that occurred [77]. Nurse-midwives justified their mistreatment of women by claiming that they were attempting to ensure safe outcomes for mothers and babies [13,59,77,87], and excused the perpetration of physical abuse as a “necessity” to ensure compliance and safe birth outcomes, believing that they were “forced by circumstance” [13,54,59,77,87]. Nurses and midwives from South Africa and Cambodia confirmed the urge to use physical aggression to deal with anger or frustration at a noncompliant woman [75,77,80]. Obstetricians from Brazil emphasized that some parturient women were aggressive or noncompliant and arrived primed for confrontations, which contributed to the misinterpretation of interactions between women and health workers [54]. Some nurses believed that they were caught up in providing clinical care and forgot to communicate with the woman to explain what they were doing [80] or that communicating to women about every procedure or exam was repetitive [72]. Midwives in Turkey and South Africa suggested that some health workers were “caught in a superiority complex” and enjoyed exerting control over patients [59,77]. Finally, some health workers believed that abuses perpetrated against women were “ritualized” and “punitive” but occurred only with a few “rotten apples” rather than with all providers [13,54,77].

#### Consequences of the mistreatment of women

Experiences of mistreatment during childbirth have far-reaching consequences for women and communities outside of the direct woman–provider interaction. Prior experiences and perceptions of mistreatment, low expectations of the care provided at facilities, and poor reputations of facilities in the community have eroded many women’s trust in the health system and have impacted their decision to deliver in health facilities in the future, particularly in low- and middle-income countries [6,8,10,13,21,52,53,61,64,70,71,77–79,82,85,90,94,96]. Some women may consider childbirth in facilities as a last resort, prioritizing the culturally appropriate and supportive care received from traditional providers in their homes over medical intervention [70,82]. These women may desire home births where they can deliver in a preferred position, are able to cry out without fear of punishment, receive no surgical intervention, and are not physically restrained [82,85].

## Discussion

This systematic review illustrates how, in many settings worldwide, women’s childbirth experiences in health facilities are marred by instances of mistreatment, including physical and verbal abuse, a lack of supportive care, neglect, discrimination, and denial of autonomy. Our findings indicate that while these various forms of mistreatment can occur at the level of the interaction between the woman and provider, a complex range of systemic failures at the levels of the health facility and health system contribute to its occurrence, including poor supervisory structures, insufficient staffing, inadequate supply chains, poor physical conditions, and policies, facility cultures, and power dynamics that systematically disempower women.

Bowser and Hill published a landscape analysis exploring evidence for disrespect and abuse in facility-based childbirth and proposed a seven-category model [12], which was designed to stimulate dialogue and an implementation research agenda, rather than provide a comprehensive review of global evidence. As such, this systematic review builds upon the work of Bowser and Hill to present a comprehensive typology to describe and understand the mistreatment of women during childbirth. We envisage this typology continuing to evolve as more research on this important topic is conducted.

While different countries, organizations, and authors have adopted different terminology (such as “obstetric violence,” “dehumanized care,” and “disrespect and abuse”) to describe the phenomenon discussed in this review, we have proposed “mistreatment of women” as a broader, more inclusive term that better captures the full range of experiences women and health care providers have described in the literature. These experiences can be active (such as intentional or deliberate physical abuse), passive (such as unintentional neglect due to staffing constraints or overcrowding), related to the behavior of individuals (verbal abuse by health care providers against women), or related to health system conditions (such as a lack of beds compromising basic privacy and confidentiality). However, they can all impact on a woman’s health, her childbirth experiences, and her rights to respectful, dignified, and humane care during childbirth.

Health system factors can be experienced directly by women as mistreatment. For example, a woman may feel that her privacy was violated in a labor ward without curtains or partitions available to allow health care providers and women the necessary privacy during vaginal examinations. Similarly, staffing shortages may mean that health care providers are unable to attend to all women during childbirth, which can be experienced directly by women as neglect. However, health system constraints may also have indirect effects as contributing factors to the behaviors of individuals. For example, staffing shortages, poor infrastructure, or lack of medications can create stressful working environments, which may predispose health care providers to behave poorly (or even abusively) towards women. It is important to note that mistreatment or abusive conduct by health care providers is not necessarily intentional, and may coexist with other compassionate and respectful care practices. However, women’s experiences of mistreatment must be viewed as such, regardless of intent. Health system factors may provide contextual explanations for negative experiences, but should not be considered as justification of the continued mistreatment of women. The conceptual model proposed by Freedman and Kruk illustrates the need to engage stakeholders at local, national, and international levels to participate in the discourse on the drivers of the mistreatment of women [28].

Increasing the proportion of women delivering with skilled birth attendants requires substantial efforts to improve quality of care. However, research has largely concentrated on issues related to the provision of clinical aspects of care; we were not able to identify any existing systematic reviews of interventions or strategies to improve women’s experiences of care during childbirth. Improvements to the quality of care need to not only ensure access to timely, safe and effective clinical care, but must also protect and promote women’s rights to dignified and respectful care [20]. The WHO quality of care framework for mothers and newborns makes explicit the need for more evidence and action on good communication, respect, dignity, and emotional support in efforts to improve the quality of care [23]. This approach could empower women, promote positive childbirth experiences, and increase satisfaction, but could also increase demand for and utilization of maternal health services [98–100].

The mistreatment of women during childbirth is not only a quality of care issue, but is also demonstrative of larger human rights violations. Every woman has the right to the highest attainable standard of health, which includes the right to dignified, respectful health care throughout pregnancy and childbirth, as well as the right to be free from violence and discrimination. Mistreatment, neglect, abuse, or disrespect during childbirth can amount to a violation of a woman’s fundamental human rights, as described in internationally adopted human rights standards and principles. In particular, women have the right “to be equal in dignity, to be free to seek, receive and impart information, to be free from discrimination, and to enjoy the highest attainable standard of physical and mental health, including sexual and reproductive health” [15,20].

### Limitations and Strengths of the Review

We were unable to differentiate between different levels of health facilities, as most primary studies did not specify the type of facility in the analysis. Different levels of health facilities have different environments that may facilitate or mitigate the mistreatment of women during childbirth. Furthermore, we did not include studies that explored mistreatment during home birth experiences, as we viewed these as conceptually different from facility-based birth experiences. The scope of this review was to synthesize research evidence (both published and from grey literature). Given the interdisciplinary scope of this topic across medicine, public health, law, and human rights domains, it is possible that relevant human rights reports or legal documentation did not meet the inclusion criteria of this review. Moreover, given the large scope of this phenomenon, it is possible that we have missed some articles that may have been relevant. Although no language filters were used in the search, it is possible that the searches did not yield articles published in non-Latin alphabets, and four studies were excluded because they were not published in English, French, Spanish, or Portuguese. However, it is unlikely that the exclusion of these studies impacted the model generated by this review or limits its global applicability.

There are several important strengths to this review. To our knowledge, this is the first systematic review of the mistreatment of women during childbirth in health facilities. Second, the typology presented in this review is designed to inform an evidence-based classification system and definition of how women are mistreated during childbirth, based on the findings of systematic mixed-methods evidence syntheses. Third, using the rigorous CERQual approach to assess the confidence in the review findings affords more credibility, reliability, and transparency to the analysis [36,37,41]. Finally, with the help of an international team of researchers, we included 65 studies published in four languages, which allowed us to conduct a comprehensive global synthesis across diverse settings.

### Implications for Future Research

This review found that there is limited availability of quantitative evidence regarding the burden of the mistreatment of women during childbirth in health facilities. Furthermore, the complex relationships between health system constraints, health care provider behavior, and women’s experiences of mistreatment need greater exploration in order to improve the quality of maternity care. Moving forward, the typology presented in this review can be used in the development and validation of indicators and tools to measure the prevalence of the mistreatment of women during childbirth, to identify interventions to reduce this mistreatment, and to inform efforts to develop global consensus on the definition of the mistreatment of women during childbirth. Such efforts are necessary not only to protect women’s fundamental human rights, but also to promote a women-centered approach to the provision and experience of quality care. Similar efforts should be undertaken to explore the mistreatment of women during other maternal health services, such as antenatal and abortion care.

### Conclusions

This systematic review presents a comprehensive, evidence-based typology of the mistreatment of women during childbirth in health facilities. Moving forward, we propose this typology of the phenomenon be used to develop measurement tools and to inform the discourse and further research on policies and programs to prevent the mistreatment of women during childbirth.

We must seek to find a process by which women and health care providers engage to promote and protect women’s participation in safe and positive childbirth experiences. A woman’s autonomy and dignity during childbirth must be respected, and her health care providers should promote positive birth experiences through respectful, dignified, supportive care, as well as by ensuring high-quality clinical care. The development of validated and reliable tools to measure the mistreatment of women during childbirth, as well as interventions to prevent mistreatment and promote respectful care, is a critical next step. Future research and interventions addressing quality care during childbirth must emphasize that high-quality of care is respectful, humanized care [5].

## Supporting Information

S1 Table Quantitative measures of mistreatment of women during childbirth in included studies. S1 Table is comprised of four tables (Tables A, B, C, and D). Tables A, B, and C present quantitative data extracted from three studies (Okafor et al. [26], Kruk et al. [25], and Sando et al. [27]) that explored disrespectful and abusive care of women during childbirth as the primary outcome. Table D presents quantitative data extracted from nine studies that indirectly explored or included an indicator that could be classified as an experience of disrespectful or abusive care of women during childbirth.
(DOCX)

S2 Table PubMed search strategy. Detailed search terms and filters applied to generate our PubMed search.
(DOCX)

S3 Table CINAHL search strategy. Detailed search terms and filters applied to generate our CINAHL search.
(DOCX)

S4 Table Embase search strategy. Detailed search terms and filters applied to generate our Embase search.
(DOCX)

S5 Table ENTREQ statement to enhance transparency in reporting a qualitative evidence synthesis. (DOCX)

S6 Table Study summaries. Summaries of the studies included in this review (authors, publication year, location, sample characteristics, data collection, data analysis, key findings, and quality assessment).
(DOCX)

S1 Text Review protocol. Systematic review protocol outlining the background, objectives, and methodology of this systematic review.
(DOCX)
