# Supplementary material for: The Mistreatment of Women during Childbirth in Health Facilities Globally: A Mixed-Methods Systematic Review
Source: PLoS Med. 2015 Jun 30;12(6):e1001847. doi: 10.1371/journal.pmed.1001847 (PMC4488322; doi:10.1371/journal.pmed.1001847)
Supplement: S1 Table — is comprised of four tables (Tables A, B, C, and D). Tables A, B, and C present quantitative data extracted from three studies (Okafor et al. [26], Kruk et al. [25], and Sando et al. [27]) that explored disrespectful and abusive care of women during childbirth as the primary outcome. Table D presents quantitative data extracted from nine studies that indirectly explored or included an indicator that could be classified as an experience of disrespectful or abusive care of women during childbirth. (DOCX) [file pmed.1001847.s001.docx]

**S1 Table: Quantitative measures of mistreatment of women during childbirth in included studies**

*S1 Table is comprised of four tables (Table A, Table B, Table C, and Table D. Tables A, Table B and Table C present quantitative data extracted from three studies (Okafor et al 2014, Kruk et al 2014, and Sando et al 2014) that explored disrespectful and abusive care of women during childbirth as the primary outcome. Table D presents quantitative data extracted from 9 studies that indirectly explored or included an indicator that could be classified as an experience of disrespectful and abusive care of women during childbirth.*

**Table A. Quantitative measures of mistreatment extracted from Okafor et al (2014) study.**

| Study | Study characteristics | Outcome measure | # of cases | Sample size (n) | Percent | Relationship to new domains of mistreatment |
| --- | --- | --- | --- | --- | --- | --- |
| Okafor II, Ugwu EO & Obi SN (2014) | Cross-sectional survey of postpartum women who accessed services for their newborns at the immunization clinic at a hospital in Nigeria | Non-confidential care | 116 | 446 | 26.00% | Lack of informed consent and confidentiality |
|  |  | Age disclosure without consent | 72 | 446 | 16.10% | Lack of informed consent and confidentiality |
|  |  | Provision of care without privacy | 28 | 446 | 6.30% | Lack of privacy |
|  |  | Medical history disclosure without consent | 8 | 446 | 1.80% | Lack of informed consent and confidentiality |
|  |  | Disclosure of HIV status without consent | 8 | 446 | 1.80% | Lack of informed consent and confidentiality |
|  |  | Non-dignified care | 132 | 446 | 29.60% | Poor rapport between women and providers |
|  |  | Blamed or intimidated during childbirth | 55 | 446 | 12.30% | Verbal abuse |
|  |  | Threatened with caesarean delivery to discourage patient from shouting | 34 | 446 | 7.60% | Verbal abuse |
|  |  | Received slanderous remarks (aspersions) from birth attendant | 24 | 446 | 5.40% | Verbal abuse |
|  |  | Scolded, shouted at, or called stupid | 19 | 446 | 4.30% | Verbal abuse |
|  |  | Non-consented care | 243 | 446 | 54.50% | Lack of informed consent and confidentiality |
|  |  | Non-consent for c-section | 20 | 446 | 4.50% | Lack of informed consent and confidentiality |
|  |  | Non-consent for episiotomy | 114 | 446 | 25.60% | Lack of informed consent and confidentiality |
|  |  | Non-consent for augmentation of labor | 42 | 446 | 9.20% | Lack of informed consent and confidentiality |
|  |  | Non-consent for shaving of pubic hair | 34 | 446 | 7.60% | Lack of informed consent and confidentiality |
|  |  | Non-consent for sterilization | 23 | 446 | 5.20% | Lack of informed consent and confidentiality |
|  |  | Non-consent for blood transfusion | 10 | 446 | 2.20% | Lack of informed consent and confidentiality |
|  |  | Physical abuse | 159 | 446 | 35.70% | Physical abuse |
|  |  | Restrained or tied down during labor | 77 | 446 | 17.30% | Physical abuse |
|  |  | Episiotomy given or sutured without anesthesia | 41 | 446 | 9.20% | Physical abuse |
|  |  | Beaten, slapped or pinched | 32 | 446 | 7.20% | Physical abuse |
|  |  | Sexually abused by health worker | 9 | 446 | 2.00% | Physical abuse |
|  |  | Detention in the health facility | 98 | 446 | 22.00% | Loss of autonomy |
|  |  | Discharge postponed until her hospital bills are paid | 76 | 446 | 17.00% | Loss of autonomy |
|  |  | Detained in the hospital until infant's bills are paid | 22 | 446 | 4.90% | Loss of autonomy |
|  |  | Abandonment/neglect of care | 130 | 446 | 29.10% | Neglect and abandonment |
|  |  | Denied companionship by the husband or close relatives | 63 | 446 | 14.10% | Lack of supportive care |
|  |  | Being left unattended in second stage of labor | 41 | 446 | 9.20% | Neglect and abandonment |
|  |  | Birth attendant failed to intervene in a life-threatening situation | 22 | 446 | 4.90% | Neglect and abandonment |
|  |  | Discrimination on the basis of specific patient attributes | 89 | 446 | 20.00% | Stigma and discrimination |
|  |  | Denial of needed attention on the basis of ethnic origin | 13 | 446 | 2.90% | Stigma and discrimination |
|  |  | Denial of needed attention because of low social class | 61 | 446 | 13.70% | Stigma and discrimination |
|  |  | Denial of needed attention because of teenage (<= 19 years) | 9 | 446 | 2.00% | Stigma and discrimination |
|  |  | Denial of needed attention because of HIV-seropositive status | 6 | 446 | 1.30% | Stigma and discrimination |

**Table B. Quantitative measures of mistreatment extracted from Kruk et al (2014) study.**

| **Study** | **Study characteristics** | **Outcome measure** | **Data collection method** | | | | **Relationship to new domains of mistreatment** |
| --- | --- | --- | --- | --- | --- | --- | --- |
| Kruk ME, Kujawski S, Mbaruku G, Ramsey K, Moyo W & Freedman LP (2014) | Cross sectional design comprised of exit interviews and follow-up surveys with the same women who delivered in any of eight health facilities in rural Tanzania. |  | Exit survey (n=1779) | | Follow-up survey (n=593) | |  |
|  |  |  | n | % | n | % |  |
|  |  | *Any experience of disrespect and abuse* | 343 | 19.48% | 167 | 28.21% | Any experience of mistreatment |
|  |  | *Specific experiences of disrespect and abuse* | | | | | |
|  |  | **Non-confidential care** | 77 | 4.39% | 36 | 6.16% | Lack of informed consent and confidentiality |
|  |  | Lack of physical privacy | 77 | 4.39% | 36 | 6.16% | Lack of privacy |
|  |  | **Non-dignified care** | 227 | 12.89% | 112 | 18.92% | Poor rapport between women and providers |
|  |  | Shouting/scolding | 153 | 8.71% | 78 | 13.18% | Verbal abuse |
|  |  | Threat of withholding treatment | 73 | 4.16% | 35 | 6.01% | Verbal abuse |
|  |  | Threatening or negative comments | 93 | 5.28% | 68 | 11.54% | Verbal abuse |
|  |  | **Neglect** | 150 | 8.53% | 92 | 15.54% | Neglect and abandonment |
|  |  | Ignored when needed help | 139 | 7.93% | 84 | 14.24% | Neglect and abandonment |
|  |  | Delivery without attendant | 68 | 3.91% | 31 | 5.31% | Neglect and abandonment |
|  |  | **Non-consented care** | 1 | 0.06% | 1 | 0.17% | Lack of informed consent and confidentiality |
|  |  | Non-consent for tubal ligation | 1 | 0.06% | 0 | 0.00% | Lack of informed consent and confidentiality |
|  |  | Non-consent for c-section | 0 | 0.00% | 1 | 0.18% | Lack of informed consent and confidentiality |
|  |  | Non-consent for hysterectomy | 0 | 0.00% | 0 | 0.00% | Lack of informed consent and confidentiality |
|  |  | **Physical abuse** | 51 | 2.90% | 30 | 5.08% | Physical abuse |
|  |  | Physical abuse (slapping, pinching, etc) | 47 | 2.68% | 30 | 5.10% | Physical abuse |
|  |  | Sexual harassment | 2 | 0.11% | 1 | 0.17% | Physical abuse |
|  |  | Rape | 4 | 0.23% | 0 | 0.00% | Physical abuse |
|  |  | Beaten, slapped or pinched | 34 | 1.94% | 20 | 3.39% | Physical abuse |
|  |  | Sexually abused by health worker | 3 | 0.17% | 2 | 0.34% | Physical abuse |
|  |  | **Inappropriate demands for payment** | 31 | 1.78% | 18 | 3.07% | Facility culture |

**Table C. Quantitative measures of mistreatment extracted from Sando et al (2014) study.**

| **Study** | **Study characteristics** | **Outcome measure** | **Data collection method** | | | | | | | | **Relationship to new domains of mistreatment** |
| --- | --- | --- | --- | --- | --- | --- | --- | --- | --- | --- | --- |
| Sando D, Kendall T, Lyatuu G, Ratcliffe H, McDonald K, Mwanyika-Sando, M, Emil F, Chalamilla G and Langer A (2014) | Mixed-methods design comprising 4 main activities: (1) quantitative interviews with postpartum women 3–6 hours after childbirth; (2) direct observation of labor; (3) quantitative interviews with providers; and (4) in-depth interviews with providers. |  | **Women's self-report** | | | | **Direct observation of labor** | | | |  |
|  |  |  | HIV positive (n=147) | | HIV negative (n=1807) | | HIV positive (n=18) | | HIV negative (n=183) | |  |
|  |  |  | n | % | n | % | n | % | n | % |  |
|  |  | *Any experience of disrespect and abuse* | 18 | 12.20% | 271 | 15.00% | - | - | - | - | Any experience of mistreatment |
|  |  | *Specific experiences of disrespect and abuse* | | | | | | | | | |
|  |  | Non-confidential care | 1 | 0.70% | 33 | 1.80% | - | - | - | - | Lack of informed consent and confidentiality |
|  |  | Lack of physical privacy | 1 | 0.70% | 36 | 2.00% | - | - | - | - | Lack of privacy |
|  |  | Woman's medical history was discussed where others could hear | - | - | - | - | 4 | 22.20% | 37 | 20.20% | Lack of informed consent and confidentiality |
|  |  | Auditory privacy was not respected during postnatal examination | - | - | - | - | 0 | 0.00% | 11 | 6.20% | Lack of informed consent and confidentiality |
|  |  | Non-dignified care | 7 | 4.80% | 117 | 6.50% | - | - | - | - | Poor rapport between women and providers |
|  |  | Provider used non-dignified language during history taking | - | - | - | - | 0 | 0.00% | 9 | 4.90% | Poor rapport between women and providers |
|  |  | Provider used a harsh tone or shouted while taking the woman's medical history | - | - | - | - | 1 | 5.60% | 12 | 6.60% | Verbal abuse |
|  |  | Bed in postnatal ward was not clean | - | - | - | - | 12 | 70.60% | 114 | 64.00% | Physical condition of facilities |
|  |  | Non-consented care | 2 | 1.40% | 3 | 0.20% | - | - | - | - | Lack of informed consent and confidentiality |
|  |  | Woman not asked for consent for first examination in antenatal ward | - | - | - | - | 16 | 88.90% | 156 | 85.30% | Lack of informed consent and confidentiality |
|  |  | Woman not asked for consent for vaginal examination in antenatal ward | - | - | - | - | 18 | 100.00% | 146 | 79.80% | Lack of informed consent and confidentiality |
|  |  | Physical abuse | 4 | 2.70% | 85 | 4.70% | - | - | - | - | Physical abuse |
|  |  | Episiotomy without anesthesia | - | - | - | - | 1 | 5.60% | 8 | 4.40% | Physical abuse |
|  |  | Woman's legs tied | - | - | - | - | 0 | 0.00% | 6 | 3.30% | Physical abuse |
|  |  | Woman's arms tied | - | - | - | - | 0 | 0.00% | 6 | 3.30% | Physical abuse |
|  |  | Detention in facility for failure to pay | 10 | 6.8 | 143 | 7.9 | - | - | - | - | Loss of autonomy |
|  |  | Request for bribe | 1 | 0.7 | 2 | 0.1 | - | - | - | - | Facility culture |
|  |  | Detention | - | - | - | - | 17 | 94.40% | 167 | 91.30% | Loss of autonomy |
|  |  | Lack of privacy | - | - | - | - | 12 | 66.70% | 119 | 65.00% | Lack of privacy |
|  |  | Partitions did not provide privacy | - | - | - | - | 17 | 94.40% | 167 | 91.30% | Lack of privacy |
|  |  | Woman's naked body exposed/not well covered during labor and delivery | - | - | - | - | 12 | 66.70% | 119 | 65.00% | Lack of privacy |

**Table D.** Quantitative measures of the mistreatment of women during childbirth in other included studies that explored this concept indirectly (Note: none of the studies included in this table used the experience of the mistreatment of women during childbirth in facilities as a primary outcome. However, several studies reported on indicators that fall into the domains of mistreatment of women as defined by the qualitative evidence synthesis. Qualitative studies that reported descriptive statistics or used a quantitative analysis method are reported in the qualitative evidence synthesis and not in this table).

| Small R, Yelland J, Lumley J, Brown S, Liamputtong P (2002) | Cross-sectional population-based survey of immigrant women who delivered in Australia, structured interviews with some open ended questions | Several times decisions were taken without my wishes being taken into account | 90 | 311 | 28.9% | Ineffective communication, loss of autonomy |
| --- | --- | --- | --- | --- | --- | --- |
|  |  | I felt my labor and/or the birth was taken over by strangers and/or machines | 84 | 288 | 20.2% | Loss of autonomy |
|  |  | Unwanted people were present at the birth | 22 | 315 | 7.0% | Loss of autonomy |
|  |  | Staff lacking in concern, rude, cold, not gentle, uncaring, hostile | 28 | 95 | 29.5% | Failure to meet professional standards, verbal abuse |
|  |  | Staff did not explain things, ignored requests | 21 | 95 | 22.1% | Ineffective communication |
|  |  | Staff did nothing to help with pain | 6 | 95 | 6.3% | Refusal to provide pain relief |
|  |  | Left alone too much in labor; felt neglected | 14 | 95 | 14.7% | Lack of supportive care, neglect and abandonment |
|  |  | Problems communicating with staff (no interpreters, couldn’t express wishes) | 12 | 95 | 12.6% | Ineffective communication |
| Chalmers B & Hashi K (2000) | Cross-sectional survey of Somali women with female genital mutilation who delivered in Canada | Hurtful comments made by their caregivers regarding their circumcision (summary category) | - | - | 87.5% | Verbal abuse |
|  |  | Verbal expressions of surprise when the perineum was seen by their doctors | - | - | 74.2% | Lack of supportive care, stigma & discrimination |
|  |  | Non-verbal expressions of surprise when  the perineum was seen by their doctors | - | - | 78.0% | Lack of supportive care, stigma & discrimination |
|  |  | Regarded with disgust | - | - | 55.1% | Verbal abuse |
|  |  | No respect shown for their cultural practice | - | - | 57.4% | Loss of autonomy |
|  |  | Touched roughly during delivery | - | - | 20.1% | Physical abuse, failure to meet professional standards |
|  |  | Doctor had not avoided exposing their perineal area unnecessarily during labor | - | - | 33.1% | Lack of privacy, objectification |
|  |  | No discussion of possible procedures or options before delivery | 345 | 432 | 79.9% | Loss of autonomy (passive participants), lack of informed consent process |
|  |  | Felt that the nurses were generally highly insensitive to their postpartum pain | - | - | 40.5% | Lack of supportive care |
|  |  | Felt that the nurses were unaware that women with circumcision experienced particularly severe postpartum pain | - | - | 13.7% | Lack of supportive care |
| Brown H, Hofmeyr GJ, Nikodem V, Smith H, & Garner P (2007) | Pilot randomized control trial of an intervention to promote birth companions in hospital deliveries in South Africa | Not allowed companion | - | 2085 | 84.5% | Lack of supportive care |
|  |  | Left alone | - | 2080 | 16.2% | Neglect and abandonment |
|  |  | Not allowed food | - | 2089 | 77.4% | Loss of autonomy |
|  |  | Not allowed fluids | - | 2089 | 83.6% | Loss of autonomy |
|  |  | Moving around not allowed during first stage of labor | - | 2083 | 46.6% | Loss of autonomy |
|  |  | Moving around not allowed during second stage of labor | - | 2072 | 97.1% | Loss of autonomy |
|  |  | Shouted at | - | 2085 | 17.7% | Verbal abuse |
|  |  | Slapped or struck | - | 2080 | 4.3% | Physical abuse |
| Silal S, Penn-Kekana L, Harris B, Birch S, McIntyre D (2012) | Mixed-methods with quantitative and in-depth interviews of women who delivered in South Africa | Felt that the health care worker was too busy | 265 | 1167 | 22.7% | Lack of supportive care, neglect and abandonment |
|  |  | Shouted at during labor | 208 | 1223 | 17.0% | Verbal abuse |

| Busanello J, da Costa P, Mendoza-Sassi R, Souza O, Gonsalves B (2011) | Cross-sectional survey of health workers in an obstetric center in Brazil on the humanization of adolescent delivery services | Disrespect to privacy and intimacy | 7 | 23 | 30.4% | Lack of privacy |
| --- | --- | --- | --- | --- | --- | --- |
|  |  | Bad or very bad relation with the parturient and family | 4 | 23 | 17.4% | Ineffective communication, lack of supportive care |
|  |  | Lack of information about delivery | 2 | 23 | 8.7% | Ineffective communication, loss of autonomy |
|  |  | Lithotomy as a routine position of delivery | 22 | 23 | 95.6% | Loss of autonomy |
|  |  | Not involved with the type of delivery | 20 | 23 | 87.0% | Loss of autonomy |
|  |  | Routine trichotomy | 16 | 23 | 69.6% | Loss of autonomy |
|  |  | Routine enema | 1 | 23 | 4.3% | Loss of autonomy, failure to meet professional standards |
|  |  | Routine episiotomy | 18 | 23 | 78.3% | Loss of autonomy, failure to meet professional standards |
|  |  | Fasting | 5 | 23 | 21.7% | Loss of autonomy |

| Nagahama E & Santiago S (2008) | Cross-sectional survey of women in Parana, Brazil to identify facilitators and barriers to the implementing humanized care | Absence of companion in the labor ward | 238 | 569 | 41.8% | Lack of supportive care |
| --- | --- | --- | --- | --- | --- | --- |
|  |  | Absence of companion on the delivery ward | 561 | 569 | 98.6% | Lack of supportive care |
|  |  | Lack of use of non-pharmacological pain killer methods | 186 | 569 | 32.7% | Failure to meet professional standards |
| Hulton L, Matthews Z & Stones R (2007) | Mixed methods study employing qualitative observations, in-depth interviews and quantitative exit surveys and community surveys in urban India | No explanation from their principal attendant about what was happening to them during labor and childbirth | - | - | 75.0% | Ineffective communication |
|  |  | Felt that care was hurried or neglectful | - | - | 10.0% | Neglect and abandonment |
|  |  | Shouted at or slapped | - | - | 9.0% | Verbal abuse, physical abuse |

| Hatamleh R, Shaban I & Homer C (2013) | Mixed methods study using structured survey and semi-structured interviews to evaluate satisfaction with childbirth services in Jordan | Did not experience politeness, courtesy, or respect from care providers and did not have an opportunity to clarify advice or information | 143 | 460 | 31.1% | Ineffective communication, lack of supportive care |
| --- | --- | --- | --- | --- | --- | --- |
|  |  | Treated as if they were a machine with no sense of individualized care and a lack of encouragement during labor and birth | 101 | 460 | 22.0% | Loss of autonomy, lack of supportive care |
|  |  | Lack of privacy or sensitivity | 60 | 460 | 13.0% | Lack of privacy, lack of supportive care |
|  |  | Health care provider had shouted at them, had neglected them, or had been unhelpful by failing to inform them about their progress in labor | 166 | 460 | 36.1% | Verbal abuse, neglect and abandonment, ineffective communication |

| Faneite J, Feo A, Merlo JT (2012) | Descriptive cross-sectional study consisting of a survey of awareness of obstetric violence among obstetrics personnel in health centers across Venezuela | Awareness of Ley Orgánica sobre el Derecho de las Mujeres a una Vida Libre de Violencia [Statute on the right of women to a life free from violence] | 199 | - | 45.7% | Health systems policies |
| --- | --- | --- | --- | --- | --- | --- |
|  |  | Aware of the term “obstetric violence” | 446 | - | 89.2% | Health system policies and culture |
|  |  | Who perpetrates obstetric violence? [summary category] | - | - | - | - |
|  |  | Obstetric violence is exerted by any health provider | 412 | - | 82.4% | Rapport between women and providers |
|  |  | Obstetric violence is exerted by  only obstetricians | 88 | - | 17.6% | Rapport between women and providers |
|  |  | Ever seen any mistreatment of a pregnant woman during their care (summary category) | 318 | - | 63.6% | - |
|  |  | Reported doctor as perpetrator | 136 | 318 | 42.8% | - |
|  |  | Reported nurse as perpetrator | 135 | 318 | 42.5% | - |
|  |  | Reported other health provider as perpetrator | 47 | 318 | 14.8% | - |
|  |  | Facility does not have means to allow women to deliver vertically | 473 | 500 | 94.6% | Lack of respect for birth position |
|  |  | What are the complaint mechanisms for obstetric violence? (summary category) | - | - | - | - |
|  |  | Don’t know complaint mechanism | 363 | 500 | 72.6% | Health system policies |
|  |  | Aware of complaint mechanism | 137 | 500 | 27.4% | Health system policies |
|  |  | Correct identification of complaint mechanisms | 82 | 137 | 59.9% | Health system policies |
|  |  | Aware of agencies responsible for providing support to women who experienced obstetric violence in Venezuela | 359 | 500 | 71.8% | Health system resources, policies and culture |
|  |  | Correct identification of agencies responsible for providing support to women who experienced obstetric violence in Venezuela | 103 | 141 | 73.0% | Health system resources, policies and culture |
|  |  | Ever sued for obstetric violence | 2 | 500 | 0.4% | Health system resources, policies and culture |
