# Supplementary material for: The Mistreatment of Women during Childbirth in Health Facilities Globally: A Mixed-Methods Systematic Review
Source: PLoS Med. 2015 Jun 30;12(6):e1001847. doi: 10.1371/journal.pmed.1001847 (PMC4488322; doi:10.1371/journal.pmed.1001847)
Supplement: S2 Table — Detailed search terms and filters applied to generate our PubMed search. (DOCX) [file pmed.1001847.s002.docx]

**S2 Table: PubMed Search Strategy**

4 September 2013

Developed by Meghan Bohren & Lori Rosman

Mistreatment of women during childbirth in facilities systematic review

|  | **#** | **Searches** | **Results** |
| --- | --- | --- | --- |
| **MATERNAL / PERINATAL HEALTH** | 1 | “obstetric delivery”[tiab] OR "obstetric deliveries”[tiab] OR "delivery, obstetric"[Mesh] | 59290 |
|  | 2 | “perinatal care”[tiab] OR “peri natal care”[tiab] OR “perinatal healthcare”[tiab] OR “peri natal healthcare”[tiab] OR “perinatal health care”[tiab] OR “peri natal health care”[tiab] OR "perinatal care"[Mesh] | 7420 |
|  | 3 | “maternal health services"[mesh] | 33049 |
|  | 4 | (“perinatal service”[tiab] OR “peri natal service”[tiab] OR “perinatal services”[tiab] OR “peri natal services”[tiab] OR “perinatal health service”[tiab] OR “peri natal health service”[tiab] OR “perinatal health services”[tiab] OR “peri natal health services”[tiab] OR “maternal care”[tiab] OR “maternal health care”[tiab] OR “maternal healthcare”[tiab] OR “maternal service”[tiab] OR “maternal health service”[tiab] OR “maternal services”[tiab] OR “maternal health services”[tiab]) AND (birth[tiab] OR births[tiab] OR childbirth[tiab] OR childbirths[tiab] OR delivery[tiab] OR deliveries[tiab]) | 932 |
|  | 5 | #1 OR #2 OR #3 OR #4 | 90527 |
| **FACILITIES** |  |  |  |
|  | 6 | “birthing centers"[tiab] OR "maternal-child health centers"[tiab] OR "delivery rooms"[tiab] OR "maternity hospitals"[tiab] | 1131 |
|  |  |  |  |
| **FACILITY-BASED DELIVERY** | 7 | “facility based delivery”[tw] OR “facility based deliveries”[tw] OR “facility delivery”[tw] OR “facility deliveries”[tw] OR “facility based births”[tw] OR “facility based birth”[tw] “facility-based childbirth”[tw] OR “facility-based child birth”[tw] OR “facility birth”[tw] OR “facility births”[tw] OR “clinic delivery”[tw] OR “clinic deliveries”[tw] OR “clinic births”[tw] OR “clinic birth”[tw] OR “hospital delivery”[tw] OR “hospital deliveries”[tw] OR “hospital birth”[tw] OR “hospital births”[tw] OR “hospital childbirth”[tw] OR “hospital childbirths”[tw] OR “hospital based deliveries”[tw] OR “hospital based delivery”[tw] OR “hospital based births”[tw] OR “institutional birth”[tw] OR “institutional births”[tw] OR “institutional childbirth”[tw] OR “institutional childbirths”[tw] OR “institutional delivery”[tw] OR “institutional deliveries”[tw] | 1259 |
|  | 8 | #5 OR #6 OR #7 | 92023 |
| **MISTREATMENT** | 9 | “disrespect”[tw] OR “disrespects”[tw] OR “disrespectful”[tw] OR “disrespected”[tw] OR “respectful”[tw] OR “abuse”[tw] OR “abused”[tw] OR “abusive”[tw] OR “abuses”[tw] OR “neglect”[tw] OR “neglected”[tw] OR “neglects”[tw] OR “confidentiality”[tw] OR “confidential”[tw] OR “non-confidential”[tw] OR “informed consent”[tw] OR “violence”[tw] OR “violent”[tw] OR “humiliation”[tw] OR “humiliate”[tw] OR “condescend”[tw] OR “condescending”[tw] OR “condescension”[tw] OR “intimidation”[tw] OR “intimidate”[tw] OR “yelling”[tw] OR “yell”[tw] OR “non dignified”[tw] OR “non-dignified”[tw] OR “undignified”[tw] OR “discrimination”[tw] OR “discriminate”[tw] OR “abandon”[tw] OR “abandonment”[tw] OR “detention”[tw] OR “human rights”[tw] OR “maltreatment”[tw] OR “mistreatment”[tw] OR “humanization”[tw] OR “humanized”[tw] OR “dehumanized”[tw] OR “dehumanization”[tw] OR “dignified”[tw] OR “undignified”[tw] OR “stigma”[tw] OR “dignity”[tw] OR “bullying”[tw] OR “bully”[tw] | 429034 |
|  | 10 | “confidentiality”[mesh] or “informed consent"[mesh] or "women's rights"[mesh] or "violence"[mesh] or "social stigma"[mesh] or "health services/ethics"[mesh] or "health care quality, access, and evaluation/ethics"[mesh] | 154108 |
|  | 11 | #8 AND (#9 OR #10) | 2999 |
