# Supplementary material for: The Mistreatment of Women during Childbirth in Health Facilities Globally: A Mixed-Methods Systematic Review
Source: PLoS Med. 2015 Jun 30;12(6):e1001847. doi: 10.1371/journal.pmed.1001847 (PMC4488322; doi:10.1371/journal.pmed.1001847)
Supplement: S3 Table — Detailed search terms and filters applied to generate our CINAHL search. (DOCX) [file pmed.1001847.s003.docx]

**S3 Table: CINAHL search strategy**

2 September 2013

Developed by Meghan Bohren & Lori Rosman

Mistreatment of women during childbirth in facilities systematic review

| **#** | **Searches** | **Results** |
| --- | --- | --- |
| S1 | (MH "Maternal-Child Care+") OR (MH “Obstetric Patients+”) OR (MH “Rooming In+”) OR (MH “Obstetric Service+”) OR (MH “Childbirth+”) OR (MH "Women's Health Services/EI") OR (MH "Obstetric Care") OR TI “perinatal care” OR TI “perinatal service” OR TI “perinatal services” OR TI “peri natal care” OR TI “peri natal service” OR TI “peri natal services” OR TI “maternal care” OR TI “maternal service” OR TI “maternal services” OR TI childbirth OR TI childbirths OR AB “perinatal care” OR AB “perinatal service” OR AB “perinatal services” OR AB “peri natal care” OR AB “peri natal service” OR AB “peri natal services” OR AB “ante natal care” OR AB “ante natal service” OR AB “ante natal services” OR AB “maternal care” OR AB “maternal service” OR AB “maternal services” OR AB childbirth OR AB childbirths | 44,301 |
| S2 | (MH "Health Facilities+") OR TI “Health Facility” OR TI “Health Facilities” OR TI “Medical Center” OR TI “Medical Centers” OR TI “Ambulatory Care Facility”  “Ambulatory Care Facilities” OR TI “Health Center” OR TI “Health Centers” OR TI “Midwifery Service” OR TI “Midwifery Services” OR TI “Nurse-Midwifery Service” OR TI “Nurse-Midwifery Services” OR TI “Nursing Service” OR TI “Nursing Services” OR TI “Obstetric Service” OR TI “Obstetric Services” OR TI “Delivery Room” OR TI “Delivery Rooms” OR TI “Nursing Unit” OR TI “Nursing Units” OR TI “Self-Care Unit” OR TI “Self-Care Units” OR TI “Health Care Facility” OR TI “Health Care Facilities” OR TI “Hospital” OR TI “Hospitals” OR TI “Patients' Room”  OR TI “Patients' Rooms” OR  TI “Regional Center” OR TI “Regional Centers” OR TI “facility based” OR AB “Health Facility” OR AB “Health Facilities” OR AB “Medical Center” OR AB “Medical Centers” OR AB “Ambulatory Care Facility” OR AB “Ambulatory Care Facilities” OR AB “Health Center” OR AB “Health Centers” OR AB “Midwifery Service” OR AB “Midwifery Services” OR AB “Nurse-Midwifery Service” OR AB “Nurse-Midwifery Services” OR AB “Nursing Service” OR AB “Nursing Services” OR AB “Obstetric Service” OR AB “Obstetric Services” OR AB “Delivery Room” OR AB “Delivery Rooms” OR AB “Nursing Unit” OR AB “Nursing Units” OR AB “Self-Care Unit” OR AB “Self-Care Units” OR AB “Health Care Facility” OR AB “Health Care Facilities” OR AB “Hospital” OR AB “Hospitals” OR AB “Patients' Room” OR AB “Patients' Rooms” OR AB “Regional Center” OR AB “Regional Centers” OR AB “facility based” | 378,340 |
| S3 | TI “facility based delivery” OR TI “facility based deliveries” OR TI “facility delivery” OR TI “facility deliveries” OR TI “facility based births” OR TI “facility based birth” OR “facility birth” OR TI “facility births” OR TI “clinic delivery” OR TI “clinic deliveries” OR TI “clinic births” OR TI “clinic birth” OR TI “hospital delivery” OR TI “hospital deliveries” OR TI “hospital birth” OR TI “hospital births” OR TI “hospital childbirth” OR TI “hospital childbirths” OR TI “hospital based deliveries” OR TI “hospital based delivery” OR TI “hospital based births” OR TI “institutional birth” OR TI “institutional births” OR TI “institutional childbirth” OR TI “institutional childbirths” OR TI “institutional delivery” OR TI “institutional deliveries” OR AB “facility based delivery” OR AB “facility based deliveries” OR AB “facility delivery” OR AB “facility deliveries” OR AB “facility based births” OR AB “facility based birth” OR “facility birth” OR AB “facility births” OR AB “clinic delivery” OR AB “clinic deliveries” OR AB “clinic births” OR AB “clinic birth” OR AB “hospital delivery” OR AB “hospital deliveries” OR AB “hospital birth” OR AB “hospital births” OR AB “hospital childbirth” OR AB “hospital childbirths” OR AB “hospital based deliveries” OR AB “hospital based delivery” OR AB “hospital based births” OR AB “institutional birth” OR AB “institutional births” OR AB “institutional childbirth” OR AB “institutional childbirths” OR AB “institutional delivery” OR AB “institutional deliveries” | 471 |
| S4 | ((S1 AND S2) OR S3) | 9,002 |
| S5 | TI “disrespect” OR TI “disrespects” OR TI “disrespectful” OR TI “disrespected” OR TI “respectful” OR TI “abuse” OR TI “abused” OR TI “abusive” OR TI “abuses” OR TI “neglect” OR TI “neglected” OR TI “neglects” OR TI “confidentiality” OR TI “confidential” OR TI “non-confidential” OR TI “informed consent” OR TI “violence” OR TI “violent” OR TI “humiliation” OR TI “humiliate” OR TI “condescend” OR TI “condescending” OR TI “condescension” OR TI “intimidation” OR TI “intimidate” OR TI “yelling” OR TI “yell” OR TI “non dignified” OR TI “non-dignified” OR TI “undignified” OR TI “discrimination” OR TI “discriminate” OR TI “abandon” OR TI “abandonment” OR TI “detention” OR TI “human rights” OR TI “maltreatment” OR TI “mistreatment” OR TI “humanization” OR TI “humanized” OR TI “dehumanized” OR TI “dehumanization” OR TI “dignified” OR TI “undignified” OR TI “stigma” OR TI “dignity” OR TI “bullying” OR TI “bully” OR AB “disrespect” OR AB “disrespects” OR AB “disrespectful” OR AB “disrespected” OR AB “respectful” OR AB “abuse” OR AB “abused” OR AB “abusive” OR AB “abuses” OR AB “neglect” OR AB “neglected” OR AB “neglects” OR AB “confidentiality” OR AB “confidential” OR AB “non-confidential” OR AB “informed consent” OR AB “violence” OR AB “violent” OR AB “humiliation” OR AB “humiliate” OR AB “condescend” OR AB “condescending” OR AB “condescension” OR AB “intimidation” OR AB “intimidate” OR AB “yelling” OR AB “yell” OR AB “non dignified” OR AB “non-dignified” OR AB “undignified” OR AB “discrimination” OR AB “discriminate” OR AB “abandon” OR AB “abandonment” OR AB “detention” OR AB “human rights” OR AB “maltreatment” OR AB “mistreatment” OR AB “humanization” OR AB “humanized” OR AB “dehumanized” OR AB “dehumanization” OR AB “dignified” OR AB “undignified” OR AB “stigma” OR AB “dignity” OR AB “bullying” OR AB “bully” | 84,711 |
| S6 | (MH "Patient Rights/EI/ST") OR (MH "Women's Rights/EI") OR (MH "Violence") OR (MH "Stigma/EI") OR (MH "Quality of Health Care/EI/TD") OR (MH "Patient Attitudes") OR (MH "Patient Centered Care") | 47,528 |
| S7 | S4 AND (S5 OR S6) | 649 |
