# Supplementary material for: The Mistreatment of Women during Childbirth in Health Facilities Globally: A Mixed-Methods Systematic Review
Source: PLoS Med. 2015 Jun 30;12(6):e1001847. doi: 10.1371/journal.pmed.1001847 (PMC4488322; doi:10.1371/journal.pmed.1001847)
Supplement: S4 Table — Detailed search terms and filters applied to generate our Embase search. (DOCX) [file pmed.1001847.s004.docx]

**S4 Table: Embase search strategy**

2 September 2013

Developed by Meghan Bohren & Lori Rosman

Mistreatment of women during childbirth in facilities systematic review

|  | **#** | **Searches** | **Results** |
| --- | --- | --- | --- |
| **MATERNAL / PERINATAL HEALTH** | 1 | 'obstetric delivery':ab,ti OR 'obstetric deliveries':ab,ti OR ‘delivery’/de | 33,963 |
|  | 2 | ‘perinatal care’:ti,ab OR ‘peri natal care’:ti,ab OR ‘perinatal healthcare’:ti,ab OR ‘peri natal healthcare’:ti,ab OR ‘perinatal health care’:ti,ab OR ‘peri natal health care’:ti,ab OR 'perinatal care'/de | 9877 |
|  | 3 | 'maternal care'/de | 11,677 |
|  | 4 | (‘perinatal service’:ti,ab OR ‘peri natal service’:ti,ab OR ‘perinatal services’:ti,ab OR ‘peri natal services’:ti,ab OR ‘perinatal health service’:ti,ab OR ‘peri natal health service’:ti,ab OR ‘perinatal health services’:ti,ab OR ‘peri natal health services’:ti,ab OR ‘maternal care’:ti,ab OR ‘maternal health care’:ti,ab OR ‘maternal healthcare’:ti,ab OR ‘maternal service’:ti,ab OR ‘maternal health service’:ti,ab OR ‘maternal services’:ti,ab OR ‘maternal health services’:ti,ab) AND (‘birth’:ti,ab OR ‘births’:ti,ab OR ‘childbirth’:ti,ab OR ‘child birth’:ti,ab OR ‘childbirths’:ti,ab OR ‘child births’:ti,ab OR ‘delivery’:ti,ab OR ‘deliveries’:ti,ab) | 941 |
|  | 5 | #1 OR #2 OR #3 OR #4 | 54,258 |
| **FACILITIES** | 6 | ‘birthing centers’:ti,ab OR ‘maternal-child health centers’:ti,ab OR ‘delivery rooms’:ti,ab OR ‘maternity hospitals’:ti,ab OR 'delivery room'/de | 3008 |
| **FACILITY-BASED DELIVERY** | 7 | ‘facility based delivery’:ti,ab OR ‘facility based deliveries’:ti,ab OR ‘facility delivery’:ti,ab OR ‘facility deliveries’:ti,ab OR ‘facility based births’:ti,ab OR ‘facility based birth’:ti,ab ‘facility-based childbirth’:ti,ab OR ‘facility-based child birth’:ti,ab OR ‘facility birth’:ti,ab OR ‘facility births’:ti,ab OR ‘clinic delivery’:ti,ab OR ‘clinic deliveries’:ti,ab OR ‘clinic births’:ti,ab OR ‘clinic birth’:ti,ab OR ‘hospital delivery’:ti,ab OR ‘hospital deliveries’:ti,ab OR ‘hospital birth’:ti,ab OR ‘hospital births’:ti,ab OR ‘hospital childbirth’:ti,ab OR ‘hospital childbirths’:ti,ab OR ‘hospital based deliveries’:ti,ab OR ‘hospital based delivery’:ti,ab OR ‘hospital based births’:ti,ab OR ‘institutional birth’:ti,ab OR ‘institutional births’:ti,ab OR ‘institutional childbirth’:ti,ab OR ‘institutional childbirths’:ti,ab OR ‘institutional delivery’:ti,ab OR ‘institutional deliveries’:ti,ab | 1455 |
|  | 8 | #5 OR #6 OR #7 | 57,601 |
| **DISRESPECT & ABUSE** | 9 | ‘disrespect’:ti,ab OR ‘disrespects’:ti,ab OR ‘disrespectful’:ti,ab OR ‘disrespected’:ti,ab OR ‘respectful’:ti,ab OR ‘abuse’:ti,ab OR ‘abused’:ti,ab OR ‘abusive’:ti,ab OR ‘abuses’:ti,ab OR ‘neglect’:ti,ab OR ‘neglected’:ti,ab OR ‘neglects’:ti,ab OR ‘confidentiality’:ti,ab OR ‘confidential’:ti,ab OR ‘non-confidential’:ti,ab OR ‘informed consent’:ti,ab OR ‘violence’:ti,ab OR ‘violent’:ti,ab OR ‘humiliation’:ti,ab OR ‘humiliate’:ti,ab OR ‘condescend’:ti,ab OR ‘condescending’:ti,ab OR ‘condescension’:ti,ab OR ‘intimidation’:ti,ab OR ‘intimidate’:ti,ab OR ‘yelling’:ti,ab OR ‘yell’:ti,ab OR ‘non dignified’:ti,ab OR ‘non-dignified’:ti,ab OR ‘undignified’:ti,ab OR ‘discrimination’:ti,ab OR ‘discriminate’:ti,ab OR ‘abandon’:ti,ab OR ‘abandonment’:ti,ab OR ‘detention’:ti,ab OR ‘human rights’:ti,ab OR ‘maltreatment’:ti,ab OR ‘mistreatment’:ti,ab OR ‘humanization’:ti,ab OR ‘humanized’:ti,ab OR ‘dehumanized’:ti,ab OR ‘dehumanization’:ti,ab OR ‘dignified’:ti,ab OR ‘undignified’:ti,ab OR ‘stigma’:ti,ab OR ‘dignity’:ti,ab OR ‘bullying’:ti,ab OR ‘bully’:ti,ab | 385,282 |
|  | 10 | 'confidentiality'/de OR 'informed consent'/de OR 'women`s rights'/de OR 'violence'/de OR 'stigma'/de OR 'social stigma'/de OR 'medical ethics'/de OR 'nurse attitude'/de OR 'physician attitude'/de OR 'patient abuse'/de OR 'physical abuse'/de OR 'sexual abuse'/de OR 'emotional abuse'/de | 270,845 |
|  | 11 | #8 AND (#9 OR #10) | 2955 |
