# Supplementary material for: The Mistreatment of Women during Childbirth in Health Facilities Globally: A Mixed-Methods Systematic Review
Source: PLoS Med. 2015 Jun 30;12(6):e1001847. doi: 10.1371/journal.pmed.1001847 (PMC4488322; doi:10.1371/journal.pmed.1001847)
Supplement: S6 Table — Summaries of the studies included in this review (authors, publication year, location, sample characteristics, data collection, data analysis, key findings, and quality assessment). (DOCX) [file pmed.1001847.s006.docx]

**S6 Table: Study summaries**

**Note:** summaries of included studies that utilized both quantitative and qualitative research methodologies only specify the qualitative components as the quantitative data was not extracted for the purposes of this review. Abbreviations: Traditional birth attendant (TBA), community health worker (CHW). (*) Study number does not refer to the number on the reference list.

| # ***** | Study (author/year) | Location | Sample characteristics | Data collection & analysis | Findings  * relevant sections regarding disrespectful and abusive care summarized by the reviewer | Quality assess-ment |
| --- | --- | --- | --- | --- | --- | --- |
| 1 | Fujita N, Perrin X, Vodounon J, et al (2012) | Porto-Novo, Benin | 16 hospital staff (midwives, nurse assistants, specialist doctors and hospital staff) | In-depth interviews  Grounded theory, thematic analysis | Humanized care was originally seen as a foreign concept and staff were initially hesitant to change their practices. However, through training and practice, midwives became comfortable with assisting deliveries in non-supine positions. Providers felt more actively involved in supporting women during pregnancy, labor and delivery. Women were now able to have a birth companion of their choice, as well as eat, drink and move around during labor. Communication between the providers and women was improved. Providing humanized care made providers feel more confident, trusted and proud. | Medium quality |
| 2 | Enderle CF, Kerber NPC, Susin LRO, & Concalves BG (2012) | Porto Alegre, Rio Grande do Sul, Brazil | 269 adolescents who recently delivered at a university hospital | Semi-structured interviews  Thematic analysis | Adolescents viewed appropriate delivery care as care that was provided with guidance and respect from the health workers. Open dialogue and active participation during labor and childbirth were critical. Adolescents desired timely attention from health workers, clear explanations of procedures and examinations. Overall, adolescents sought to be active participants in the labor and delivery process and positive interactions with health workers. | Medium quality |
| 3 | Redshaw M & Hockley C (2010) | England | 2,960 women who were 3 months postpartum, as identified by birth registration records | Mailed survey including open ended questions. Thematic analysis of open ended questions | Women reported that providers dismissed their concerns during labor, including those with pre-existing health conditions. Ineffective communication continued as providers did not communicate the need for intervention or surgery to the women. Women felt that they were passive participants who only had the illusion of choice during labor and delivery. Women felt alone and neglected during their deliveries, like they were “just another new mother” to the providers. | Medium quality |
| 4 | Kruger L & Schoombee C (2010) | Semi-rural community in South Africa | 93 women, 8 maternity ward nurses | In-depth interviews  Grounded theory, thematic analysis | Abusive maternity care was a prominent theme and both women and providers suggested that such abuse has become “ritualised, sanctioned, normalised and ultimately institutionalised”. Nurses were systematically disempowered by the hierarchical health system while patients were referred to as “docile passive bodies” on the maternity wards. As nurses struggled to stay in control of the maternity wards, the environment and their behavior led both the nurses and their patients to feel disappointed, frustrated and resentful. In order for disrespectful and abusive treatment on maternity wards to end, nurses and patients must be empowered as critical players in the health system. | High quality |
| 5 | Turan J, Miller S, Bukusi E, Sande J, Cohen C (2008) | Kisumu, Kenya | 17 maternity workers, 14 women, 4 male partners, 2 TBAs  22 birth observations | In-depth interviews and birth observations  Thematic analysis | HIV-related fears play an important role in where women deliver and difficulties facing maternity workers in caring for HIV positive women. Women feared being forced to test for HIV or tested for HIV without their consent, as a positive HIV test was viewed as a death sentence. Women also feared unwanted disclosure of HIV status by providers during maternity care to other patients or a male partner. Maternity workers confirmed that protecting confidentiality of HIV status was difficult on crowded maternity wards. Health workers did not report segregating HIV-positive women during care or other discriminatory practices. | High quality |
| 6 | Busanello J, da Costa P, Mendoza-Sassi R, Souza O, Gonsalves B (2011) | Porto Alegre, Rio Grande do Sul, Brazil | 23 health workers (nurse, nurse assistant, doctors, students) | Semi-structured interviews, medical record review  Chi-square, Fisher’s exact | Health workers tended to believe that privacy and autonomy of women was preserved during childbirth. However, many women believed that health workers did not maintain privacy (30.4%) and were not involved in the type of delivery selected (87.0%). Furthermore, women were often not allowed choices regarding their care, including routine deliveries in lithotomy position (95.6%), routine trichotomy (69.6%), and routine episiotomy (78.3%). Some women were required to fast during labor (21.7%). | Low quality |
| 7 | Dzomeku M (2011) | Sekyere-West district, Ghana | 12 women delivering at the district maternity unit | In-depth interviews and focus group discussions | Women reported that negative provider attitudes influenced their usage or non-usage of the facility, as well as late arrival to the facility. Women reported not receiving explanations about procedures, being yelled at and neglect. | Low quality |
| 8 | Gao Y, Barclay L, Kildea S, Hao M, & Belton S (2010) | Rural Shanxi Province, China | 30 women aged 21-39 years | In-depth interviews  Content analysis, thematic analysis | Women feared the hospitals due to their dissatisfaction with previous experiences of facility-based delivery. They felt that staff had negative attitudes, neglected on the wards, were unable to have birth companions and feared unnecessary medical intervention. Women preferred delivering at home, where traditional birth attendants provided continuous supportive care. | Low quality |
| 9 | Small R, Yelland J, Lumley J, Brown S, Liamputtong P (2002) | Vietnamese, Turkish and Filipino immigrants in Australia | 318 women | Survey  Thematic analysis of open-ended questions, Mantel-Haenszel weighted odds ratios | Women expected to receive care that was kind, safe, supportive and respectful, and when care did not meet these standards, women were distressed. Women were dissatisfied with their overall experience when they perceived staff as unhelpful and uncaring and when they were removed from the decision-making process. Many women felt that they were not actively involved in decision-making (28.9%) and that their labor was taken over by strangers or machines (20.2%). Women also felt that staff were inconsiderate, rude, cold, not gentle, uncaring and hostile (29.5%). | Medium quality |
| 10 | Teixeira N, Pereira W (2006) | Suburbs of Cuiaba, Mato Grosso, Brazil | 10 women | In-depth interviews  Thematic analysis | Institutional culture promotes violence and disrespect on the obstetric wards. Providers were viewed as hostile and impatient during vaginal deliveries and often did not provide effective communications. Women were called derogatory names and in one case, a woman was physically tied to the bed during labor. | Low quality |
| 11 | Hulton L, Matthews Z & Stones R (2007) | Urban India | 14 providers, 70 women | Exit interviews, participant observation and in-depth interviews  Thematic analysis and prevalence | Women reported experiencing unnecessary procedures and lack of privacy during facility-based deliveries, as they often labored in public places. They felt that their care was hurried and neglectful. Women reported being shouted at and slapped during labor (9%). Women were not allowed birth companions, and felt that they lacked supportive care. | Low quality |
| 12 | El-Nemer A, Downe S, & Small N (2006) | Egypt | 21 women | In-depth interviews  Grounded theory, content analysis | Women favored home birth compared to facility birth, and desired care from providers who were “helping from the heart”. They felt that childbirth in hospitals was systemized, including lack of choice of delivery position, provision of unwanted drugs and lack of privacy. Health workers did not communicate effectively with women and were described as having a “technical touch”. They felt disempowered from their childbirth process, neglected and alone. Such experiences made some women claim that they would not return to that hospital again. | High quality |
| 13 | Oliviera Z, Madeira A (2002) | Belo Horizonte, Minas Gerais, Brazil | 8 women | In-depth interviews  Ideographic analysis | Adolescents felt that their bodies were controlled by providers during their childbirth. They were not allowed to cry out in pain. Vaginal examinations were not clearly explained by the providers and not understood by the patients, who found them to be painful. They were not allowed to choose their own delivery position. | Low quality |
| 14 | Afsana K, Rashid S (2001) | Rural Bangladesh | 41 women, 5 TBAs, 4 physicians, 7 paramedics | In-depth interviews, FGDs, birth observations  Thematic analysis | Women felt that providers in the health facilities did not communicate as effectively as TBAs. Women were not allowed to choose their delivery position, which was a key barrier to accessing facility-based care. Government staff were perceived as rude, unhelpful and slow. Privacy was not maintained in the facilities, which made women feel shameful. Health workers often shouted at women during delivery. | High quality |
| 15 | Coyle K, Hauck Y, Percival P, Kristjanson L (2001) | Western Australia, Australia | 17 women | In-depth interviews  Grounded theory, thematic analysis | Women felt that they could not build a rapport with their providers and felt that this had a negative impact on their perceptions of care. They felt that care was systemized and providers did not provide individualized care and did not communicate effectively. | Medium quality |
| 16 | Miller S, Cordero M, Coleman A, Figueroa J, Brito-Anderson S, Dabagh R, Calderon V, Caceres F, Fernandez A, Nunez M (2003) | Dominican Republic | 57 prenatal women, 55 women in labor, 21 women with vaginal delivery, 6 cesarean delivery  88 providers of maternity services | Observations and in-depth interviews.  Does not specify analysis methods | The facilities included in the observations were overcrowded and understaffed. Staff were dangerously inexperienced and higher-level providers were not seen caring for patients. Women shared beds while they were in labor and were exposed to other patients during this time. There was no privacy on the wards and women’s dignity was not maintained. Facilities were filthy with trash, bodily fluids, needles and glass visible on the beds and floor. Women were made to walk barefoot across the floor for examination. Providers had poor communication with women. Women felt neglected and abandoned during labor. | Low quality |
| 17 | Kyomuhendo G (2003) | Hoima district, Uganda | Qualitative: 240 women and men  Quantitative: 808 women (not relevant to D&A) | Focus group discussions and survey  Does not specify analysis methods | The use of facilities for childbirth was considered as a last resort, due to lack of skilled staff, abuse, neglect and poor treatment. Women had to deliver in a supine position but preferred the traditional kneeling position. Health workers rushed through the delivery of care and did not support or communicate effectively with women. Women felt abandoned during critical moments of their labor and were often asked to pay bribes. | Low quality |
| 18 | Richard F, Filali H, Lardi M, De Brouwere V (2003) | Tetouan and Sidi Kacem, Morocco | 94 women who had a severe maternal morbidity, 91 family members, 53 health staff | Semi-structured interviews, focus group discussions  Thematic analysis | Overall, women reported satisfaction with their care, which they viewed as life-saving. However, many women and their families reported mistreatment during their deliveries in the health facility. This mistreatment included poor staff attitudes, verbal abuse, having to pay bribes, lack of empathy and discrimination against women of low socioeconomic status. | High quality |
| 19 | Chalmers B, Omer-Hashi K (2002) | Ontario, Canada | 432 Somali women who had given birth to a baby in Canada | Quantitative survey  Thematic analysis of the open ended survey questions | Women with prior experience of female genital cutting (FGC) who had childbirth experiences in Canada were faced with culturally insensitive and harsh treatment from their health providers. In the facility-setting, they did not receive confidential or private care. Providers frequently made judgmental comments regarding their Muslim faith, sexual experiences and the experience of FGC. Women received ineffective communication regarding procedures and examinations. | Medium quality |
| 20 | Chalmers B, Omer-Hashi K (2000) | Ontario, Canada | 432 Somali women who had given birth to a baby in Canada | Quantitative survey  Prevalence of childbirth experiences | Women frequently experienced hurtful comments made by their caregivers regarding their circumcision (87.5%). Women were touched roughly during delivery (20.1%) and were often exposed to others (33.1%). Most women were not engaged in a discussion regarding possible procedures or options before delivery (79.9%). | Medium quality |
| 21 | Esposito NW (1999) | New York City, USA | 29 women, 5 midwives, 6 staff members at a birthing center | Ethnography, interviews and observation  Thematic analysis | Women’s contrasting experiences of childbirth in a hospital compared to a birthing center in New York City are presented. Marginalized women experienced gender, race and class-power inequities in the hospital settings, which did not meet their needs for respect and autonomy during childbirth. Women did not play an active role in their childbirth experience and did not receive supportive delivery care. They were often faced with racism and prejudice. | Medium quality |
| 22 | Brown H, Hofmeyr GJ, Nikodem VC, Smith H, Garner P (2007) | Facilities within a 200 km radius of Johannesburg, South Africa | 10 health facilities  Baseline: 2090 women  Endline: 2058 women | Quantitative survey  Change from baseline to follow-up at each study site. | Women were often not allowed to have a companion (84.5%) and felt that they were left alone during labor and delivery (16.2%). Women were usually not allowed food (77.4%) or drinks (83.6%) during labor, and were often not allowed to move around during the first (46.6%) or second (97.1%) stage of labor. Some women were shouted at (17.7%) and slapped or struck (4.3%) by their providers. | High quality |
| 23 | Beck CT (2004) | Global (Internet-based recruitment) | 40 women total from New Zealand (23), the United States (8), Australia (6) and the United Kingdom (3). Members of the Trauma and Birth Stress trust. | Birth trauma stories written by participants  Descriptive phenomenology | Four main themes were reported in the analysis: (1) to care for me: was that too much to ask? (2) to communicate with me: why was this neglected? (3) to provide safe care: you betrayed my trust and I felt powerless; and (4) the end justifies the means: at whose expense? at what price? | Medium quality |
| 24 | Janevic T, Sripad P, Bradley E, Dimitrievska V (2011) | Belgrade, Serbia and Skopje, Macedonia and two rural areas in each country | 71 women who delivered in the past year and lived in a Romani settlement, 8 gynecologists, 11 key informants | Focus group discussions and in-depth interviews  Grounded theory | Romani women perceived racism and discrimination to play a critical role in the inequalities in access to and utilization of the maternal health care system. Three types of racism (institutional, personally-mediated, and internalized) interacted with the emergent themes. Six main domains emerged in the analysis: social environment and resources, health system accountability, financial issues, education, perceptions and interactions with the health system and psychological factors. | High quality |
| 25 | Mselle LT, Kohi TW, Mvungi A, Evjen-Olsen B, Moland KM (2011) | Dar es Salaam and Mwanza, Tanzania | 16 women of reproductive age who were affected by obstetric fistula | Mixed-methods: in-depth interview and quantitative survey (not relevant)  Thematic analysis | Women with obstetric fistula experienced delays before arriving at the facility for childbirth and after arriving at the facility. Upon arrival at the facility, women faced serious delays as they were neglected, left to labor unsupervised, received inadequate labor monitoring and a lack of supportive care from providers. When faced with labor complications, women also faced significant delays in referral to higher level facilities. | High quality |
| 26 | Jomeen & Redshaw (2013) | United Kingdom | 219 ethnic minority women who recently delivered in the United Kingdom | Open-ended responses of a quantitative survey  Thematic analysis | In general, women’s expectations of receiving high quality compassionate care were not met, as they were faced with a lack of supportive care and neglect during childbirth. Women felt that they were left alone for inappropriately long periods during their labor. Providers often failed to explain procedures and claimed that they were too busy to communicate with women. Women felt that they were not provided choice in their care and sometimes did not provide informed consent. | Medium quality |
| 27 | Bangser M, Mehta M, Singer J, Daly C, Kamugumya C, Mwangomale A (2011) | 3 districts in Tanzania and 4 districts in Uganda | 137 women of reproductive age who were affected by obstetric fistula | In-depth interviews  Thematic analysis | Delays in seeking and accessing care, combined with health system delays and failures contributed to fistula experiences. Abuse and neglect by health workers, including verbal abuse, neglect and blame for their health condition, were identified as negative care experiences. In Tanzania, nurses requested bribes prior to providing care. | Medium quality |
| 28 | Cindoglu D & Sayan-Cengiz F (2010) | Ankara, Istanbul and Izmir, Turkey | 9 groups of women who ever visited health facilities for reproductive problems, 4 groups of physicians, 1 group of midwives | Focus group discussions  Thematic analysis | Although most Turkish women use hospital-based care during childbirth, many have unpleasant experiences of medicalization and patriarchy during their deliveries. Women reported nurses scolding them during labor, making condescending comments, neglect during labor, and a lack of privacy during examinations. Providers indicated that they felt that they preferred patients who fully submitted to them and felt that they were at the top of the hierarchy. | Low quality |
| 29 | Roost M, Jonsson C, Liljestrand J, Essen B (2009) | La Paz district, Bolivia | 30 women who experienced near miss event upon arrival at hospital | Semi-structured interviews  Modified analytic induction (thematic analysis) | Women who experienced near miss events perceived themselves as being fundamentally different from women who utilized health care. These women were distrustful of the healthcare system, feared mistreatment by staff and believed that they would not receive proper communication from their provider about their condition, including providing informed consent. | High quality |
| 30 | Nagahama EEI & Santiago SM (2008) | Maringa, Parana, Brazil | 569 women | Cross-sectional survey  Exploratory and descriptive analysis | Women reported that health staff had authoritarian attitudes towards their patients, which subjected women to losing autonomy over their childbirth experiences. Women found it difficult to communicate with their providers. Health staff were resistant to allowing women a male birth companion. In total, 41.8% of women had no companion on the antenatal ward and 98.6% of women had no companion on the delivery ward. | Medium quality |
| 31 | Mselle LT, Moland KM, Mvungi A, Evjen-Olsen B, Kohi TW (2013) | Dar es Salaam, Temeke & Mpwapwa districts, Tanzania | 16 women who experienced obstetric fistula, 5 nurse-midwives, 1 group of community members, 1 group of husbands | In-depth interviews and focus group discussions  Thematic analysis | Both women and their husbands and health providers experienced poor quality of care during childbirth and challenging working conditions. In particular, women reported experience of neglect, physical abuse, verbal abuse and lack of supportive care. Nurse-midwives struggled to provide quality care as they lacked supportive supervision, motivation and critical medical supplies. | Medium quality |
| 32 | Rahmani Z and Brekke M (2013) | Kabul and Ghazni provinces, Afghanistan | 12 women, 7 doctors, 5 midwives, 3 TBAs | In-depth interviews  Phenomenological analysis | Women reported dissatisfaction with childbirth services, particularly the poor attitudes and behavior of health workers, including discrimination, neglect, and verbal and physical abuse. Despite negative experiences with the health services, women appreciated having any access to health services. Health workers reported that low salaries, high stress and poor working conditions contributed to the poor quality of care. | Medium quality |
| 33 | Hatamleh R, Shaban IA, Homer C (2013) | Jordan | 460 women of reproductive age | Exit interviews  Descriptive and thematic analysis | Women reported that providers ignored and neglected them during labor and delivery, which in some cases led to women delivering in a facility without skilled attendance. Health workers did not communicate processes or procedures to women and verbally abused women. Women were not allowed to have birth companions and felt that they delivered without any human support. | Low quality |
| 34 | Ith P, Dawson A, Homer CSE (2013) | Cambodia | 30 women who delivered in health facilities | In-depth interviews  Thematic analysis | Women reported that their perception of staff attitudes and safety influenced their choice of health facility for delivery. Negative staff attitudes and lack of supportive care during labor impacted the utilization of public maternity hospitals. Women felt that they were abandoned during labor by midwives who were rude and judgmental. Health providers did not effectively communicate procedures to women and facilities were unable to provide privacy for women. | High quality |
| 35 | Kumbani LC, Chirwa E, Malata A, Odland JO, Bjune G (2012) | Chiradzulu district | 14 women of reproductive age | In-depth interviews  Thematic analysis | Women reported unsatisfactory care during their delivery in a facility, which included poor provider attitudes, delays in providing care and neglect. Health providers sometimes shouted at and ridiculed women while they were in labor, and threatened them with poor health outcomes. | High quality |
| 36 | Silal SP, Penn-Kekana L, Harris B, Birch S, McIntyre D (2012) | South Africa | Qualitative: 16 women who recently delivered  Quantitative: 1200 women who recently delivered | Mixed-methods, only qualitative relevant to review. In-depth interviews  Thematic analysis | Women reported that negative interactions with providers inhibited their access to quality maternity care. Women may fear poor treatment by health workers and choose to deliver at home or have significant delays in reaching care. Women reported that health providers verbally abused them and did not communicate clinical processes or procedures with them. While women acknowledged that health workers are overworked and underpaid, they felt that these conditions should not impact the quality of care that they receive. | High quality |
| 37 | Forssen ASK (2012) | Sweden | 20 elderly women who delivered between 1934 and 1966 | In-depth interviews  Phenomenological analysis | Traumatic birth experiences where women faced abusive care and violations of their dignity impacted women throughout their life span. Women reported that health workers blamed women for poor health outcomes and neglected them during labor. Women felt that they suffered a loss of autonomy as they were not involved in the decision-making process, had non-consented procedures and were denied pain relief. | High quality |
| 38 | Garcia-Jorda D, Diaz-Bernal Z, Alamo MA (2012) | Havana, Cuba | 36 women, 10 companions and 9 doctors | In-depth interviews and participant observation  Grounded theory | Women reported feeling a lack of supportive care during childbirth. Health providers used racial slurs when speaking to women. Women were not allowed to choose their own birth position and felt that their births were over-medicalized. | Medium quality |
| 39 | McMahon SA, George AS, Chebet JJ, Mosha IH, Mpembeni RN, Winch PJ (2014) | Morogoro region, Tanzania | 112 total participants:  49 women, 27 male partners, 20 CHWs, 5 community leaders, 11 religious leaders | In-depth interviews  Grounded theory, thematic analysis, comparison to existing frameworks | Women initially described their birth experiences in facilities in a positive light, but when probed more deeply, provided examples of mistreatment. The following themes related to disrespectful and abusive care were generated from the analysis: (1) feeling ignored or neglected; (2) monetary demands or discriminatory treatment; (3) verbal abuse; and (4) physical abuse. In response to this mistreatment, women tended to use acquiescence or non-confrontational strategies: resigning oneself to abuse, returning home, or bypassing certain facilities or providers. In contrast, male partners tended to use more assertive approaches: requesting better care, paying bribes, making complaints and assaulting a provider. | High quality |
| 40 | Faneite J, Feo A, Merlo JT | Venezuela | 500 total participants:  158 obstetricians, 115 nurses, 113 obstetric residents, 48 general practitioners, 66 other | Quantitative survey | This study explored the level of knowledge of obstetric violence and legal implications among health providers working in Venezuela. Most providers (89.2%) were aware of the term obstetric violence. The majority of providers (63.6%) had witnessed obstetric violence perpetrated by doctors (42.8%) and nurses (42.5%). Providers were familiar with the term obstetric violence, but not necessarily of the laws governing it, specific acts of obstetric violence or reporting mechanisms. | Medium quality |
| 41 | Fonn S & Philpott H (1995) | Johannesburg area, South Africa | 146 women, unspecified number of doctors and nurses | Group discussions and interviews  Thematic and descriptive analysis | Women reported negative experiences with patient-provider interactions and the resources and structure available at the facilities. Health workers reportedly hit, shout and insult women during labor. Nurses were inattentive to women’s needs and women were left alone during labor. Facilities were dirty, lacked privacy and often ran out of supplies. Women were asked to clean the linens and floors after their own deliveries. | Low quality |
| 42 | Davies MM & Bath PA (2001) | Northern England | 13 Somali women living in the United Kingdom | Semi-structured interviews and focus group discussions  Thematic analysis | Poor communication between health workers and non-English speaking Somali women was a significant barrier to seeking and understanding the childbirth process. Women consequently felt that they were denied information from their providers and believed that health workers were prejudiced against Somalis. Women disliked when their family members acted as interpreters due to privacy and confidentiality issues. | Medium quality |
| 43 | Kowalewski M, Jahn A & Kimatta SS (2000) | Mtwara region, Tanzania | 60 pregnant women, 26 health workers and 6 key informants | Semi-structured interviews  Content analysis | Women reported discrimination by health workers during maternity services. In particular, women from rural areas feared discrimination based on their socioeconomic status. Women felt neglected during their labor and felt that they lost autonomy over their childbirth experience. | Medium quality |
| 44 | Jewkes R, Abrahams N & Mvo Z (1998) | Western Cape, South Africa | 32 women, 9 midwives, 3 nurses, 1 family planning advisor, 4 group discussions | In-depth interviews and focus group discussions  Ethnographic analysis | Women reported verbal and physical abuse and neglect during childbirth in facilities. Women described nurses’ treatment of them as a few “rotten apples” but further analysis demonstrated that many other factors, including organizational, work place and power dynamics, contributed to the mistreatment of women. Nurses appeared to be struggling to create a social distance from their patients and mistreated women as a way to gain control. | Medium quality |
| 45 | Saizonou J, Godin I, Ouendo EM, Zerbo R, Dujardin B (2006) | Benin | Qualitative: 42 women who experienced near miss  Quantitative: 557 women who experienced near miss | In-depth interview, quantitative interview (not relevant to review)  Thematic analysis | In general, women reported feeling satisfied with their maternity services. However, women reported discrimination, lack of supportive care and long delays in receiving care. Facilities tended to be dirty and were unable to provide privacy to women during labor. | Medium quality |
| 46 | Atuyumbe L, Mirembe F, Johansson A, Kirumira EK, Faxelid E (2005) | Wakiso district, Uganda | 44 adolescents who were pregnant or mothers, 6 key informants | Focus group discussions and in-depth interviews  Thematic analysis | Adolescent women perceived facility-based health workers to be unfriendly, rude and have negative attitudes. Health workers made judgmental comments to adolescents regarding their sexual behavior. Adolescents perceive cesarean section as a punishment from the health worker for having a sexual relationship. Furthermore, confidentiality was not maintained during their care. | Low quality |
| 47 | Hassan SJ, Sundby J, Hhusseini A, Bjertness E (2012) | Occupied Palestinian Territory | 176 Palestinian women | Interviews with open ended questions  Descriptive and content analysis | Women reported frequent and painful vaginal examinations during labor. While most women were informed that they would have a vaginal examination, few providers explained the examination and ways to cope with the exam. Women felt that the vaginal examinations were rushed, were not conducted privately and did not follow an appropriate informed consent process. | Low quality |
| 48 | Izugbara CO & Ngilangwa DP (2010) | Slums of Nairobi, Kenya | 10 groups of women, 2 groups of TBAs, 12 women with pregnancy complications | Focus group discussions and in-depth interviews  Thematic analysis | Poor women residing in slums in Nairobi associated their poverty with poor quality of maternity care and discriminatory treatment by providers. Although many of these women used TBAs, those who delivered in a health facility expected poor treatment, including neglect and abandonment during labor. They felt that if they were unable to pay bribes, they would receive poor treatment. | High quality |
| 49 | Dietsch E, Shackleton P, Davies C, McLeod M, Alston M (2009) | Rural New South Wales, Australia | 42 women who traveled at least 1 hour from a rural community to deliver | In-depth interviews  Thematic analysis | Women felt that midwives were in a position of power and abused their authority over parturient women through threatening and aggressive behavior. Women felt alienated from their care providers as their concerns were dismissed by midwives and they were neglected while in labor. Aboriginal women felt that the midwives discriminated against them and treated them worse than other women on the ward. | Low quality |
| 50 | Magoma M, Requejo J, Campbell OMR, Cousens S, Filippi V (2010) | Ngorongoro district, Tanzania | 12 key informants, 3 groups of providers, 6 groups of women, 3 groups of TBAs, 3 groups of elder men | Focus group discussions and in-depth interviews  Grounded theory | Communication between providers and women was insufficient to explain the importance of certain procedures, such as vaginal examinations, which women viewed as painful and dehumanizing. Providers reported that other colleagues verbally abuse women. | High quality |
| 51 | Human Rights Watch (2011) | Eastern Cape, South Africa | 157 women, 30 nurses, 4 emergency medical services staff and unspecified number of key informants | In-depth interviews  Content analysis using human rights framework | Women reported severe abuses during maternity care in public health facilities. Mistreatment included physical and verbal abuse, neglect, abandonment, long delays in receiving care, discrimination and ineffective communications. Women were also asked to clean up after themselves after delivery. A lack of accountability and oversight mechanisms contributed to the inability to monitor health system performance. | Medium quality |
| 52 | Moyer CA, Adongo PB, Aborigo RA, Hodgson A, Engmann CM (2014) | Kassena-Nankana district, Ghana | 7 focus groups and 43 interviews with 128 community members (women, TBAs, heads of household, leaders, grandmothers) | Focus group discussions, in-depth interviews  Thematic analysis | Mistreatment of women during childbirth in facilities emerged as a natural theme in the focus groups and interviews. Users reported physical abuse, verbal abuse, neglect, discrimination based on socioeconomic status, denial of traditional practices and lack of choice of birth position. Women felt that they were at the bottom of the social hierarchy. | High quality |

| 53 | Oyerinde K, Harding Y, Amara P, Garbrah-Aidoo N, Kanu R, Oulare M, Shoo R, Daoh K (2013) | Sierra Leone | 4 groups of young women, 4 groups of adult women, 4 groups of young men, 4 groups of adult men, 4 groups of TBAs | Focus group discussions  Thematic analysis | Perceptions of poor health facility infrastructure, staffing constraints, supply constraints and lack of constant supportive care were significant barriers for women to deliver in facilities. Long wait times in the facilities sometimes led women to seek care from traditional providers. Women felt that they did not receive supportive care from facility-based providers, in contrast to the care they received from TBAs. | Medium quality |
| --- | --- | --- | --- | --- | --- | --- |
| 54 | D’Ambruoso L, Abbey M, Hussein J (2005) | Suburbs of Accra, Ghana | 21 women, 2 groups of women | In-depth interviews and focus group discussions  Thematic analysis | Women expected professional, respectful care during childbirth; in the absence of respectful care, women will change their place of delivery in the future. Women reported that health workers were impatient and neglected them during labor. They were often not able to consume food or drinks while on the antenatal ward. Nurses threatened women with cesarean section and demanded that women clean up after their deliveries. | High quality |
| 55 | Grossman-Kendall F, Filippi V, De Koninck M, Kanhonou L (2001) | Cotonou & Ouidah, Benin | 19 women who delivered in a referral hospital | In-depth interviews | Women reported mistreatment and humiliation by health workers, which included judgmental comments by health workers about sexual behavior and physical abuse. Poor communication and lack of consent about processes and procedures disempowered women. Women reported forceful detainment in facilities if they were unable to pay their hospital bills. | Medium quality |
| 56 | Aguiar JM, d’Oliveira AFP, Schraiber LB (2013) | Sao Paulo, Brazil | 21 women, 10 obstetricians, 5 nurses, 3 nurse technicians | In-depth interviews  Thematic analysis | In Brazil, there are blurred boundaries between exerting medical authority and violence. Health workers explained how they used jokes, threats and harsh language to gain compliance from women during delivery. However, women perceived these “jokes” as verbal abuse and threats against their well-being. Health workers reported that working with poor women was a challenge as they were prone to be difficult and not prepared for their deliveries. Using an authoritative tone was, therefore, a coping mechanism for health workers to gain patient compliance. | Low quality |
| 57 | Chadwick RJ, Cooper D, Harries J (2014) | Cape Town, South Africa | 33 low-income women of reproductive age who delivered in a public hospital | In-depth interviews  Thematic analysis | Women’s birth experiences in public hospitals in South Africa were riddled with “narratives of distress” in relation to the poor quality of intrapartum care. Four main themes emerged: (1) negative interpersonal relations with caregivers; (2) lack of information; (3) neglect and abandonment; and (4) the absence of a labor companion. | Medium quality |
| 58 | Crissman HP, Engmann CE, Adanu RM, Nimako D, Crespo K, Moyer CA (2013) | Akwatia Ghana | 85 pregnant women | In-depth interviews  Grounded theory | Ghanaian women attending antenatal care reported that harsh treatment by facility-based midwives was an important barrier to utilizing health facilities for childbirth. In particular, women reported hearing stories regarding verbal abuse from rude and impatient midwives. These women felt that they would do everything in their power to deliver in a facility, but viewed this mistreatment as a significant barrier to facility deliveries for women with home births. | Medium quality |
| 59 | Silan V, Kant S, Archana S, Misra P, Rizwan S (2014) | Dayalpur, Faridabad, Haryana, India | 5 groups of female Accredited Social Health Activists (ASHAs) | Focus group discussions  Thematic analysis | In rural Haryana government health facilities, poor quality care and mistreatment by health workers constituted significant barriers to underutilization of services. Labor wards were dirty, lacked privacy and had substantial supply and staffing constraints. Women with poor experiences in these facilities during previous births were unlikely to use these services again in the future. | Low quality |
| 60 | Sando D, Kendall T, Lyatuu G, Ratcliffe H, McDonald K, Mwanyika-Sando, M, Emil F, Chalamilla G and Langer A (2014) | Dar es Salaam, Tanzania | (1) 2000 post-partum women 3–6 hours after childbirth  (2) 208 women observed in labor  (3) 50 providers interviewed in study facility  (4) 18 providers interviewed in study facility | Mixed-methods design comprised of 4 main activities: (1) quantitative interviews with women;  (2) direct observation of labor;  (3) quantitative interviews with providers; and  (4) in-depth interviews with providers. | Overall, WLWH who received labor and delivery services at a large urban hospital in Tanzania were no more or less likely to report any type of disrespect and abuse during labor and delivery than HIV-negative women. However, many women, regardless of HIV-status, reported experiencing disrespect and abuse in the post-partum interviews. Direct observations of labor supported women’s experiences of disrespect and abuse during childbirth. Healthcare providers are aware that HIV-positive women may have more concerns about maintaining confidentiality during childbirth, but denied stigma and discrimination against them during intrapartum care. | Low quality |
| 61 | Kruk ME, Kujawski S, Mbaruku G, Ramsey K, Moyo W & Freedman LP (2014) | Rural, northeast Tanzania | Women who delivered in any of eight study facilities and were 15 years of age or older | Cross sectional design comprised of exit surveys of post-partum women, and follow-up survey with a subset of the same women 5 to 10 weeks postpartum | In the exit survey, 19% of women sampled reported experiencing at least one form of disrespectful or abusive treatment during childbirth. In the follow-up survey with a subset of the same women, 28% of women sampled reported experiencing at least one form of disrespectful or abusive treatment during childbirth. The most commonly reported experiences of mistreatment were being ignored when they needed help, shouting and scolding and negative comments. Between 3% and 5% of women reported being slapped or pinched and 4 to 5% of women reported delivering alone without a birth attendant. | Medium quality |
| 62 | Okafor II, Ugwu EO & Obi SN (2014) | Enugu state, Nigeria | Women who delivered < 6 weeks ago in Enugu State University Teaching Hospital Parklane, who accessed newborn services at the immunization clinic at the same hospital. | Cross sectional design comprised of interviews with post-partum women | Women in this sample commonly reported experiences of disrespectful and abusive care during childbirth in this facility. The most commonly reported experience was non-consented care (54.5%), including non-consent during episiotomy, augmentation of labor, sterilization, caesarean section, shaving of the pubic hair, and blood transfusion. Approximately 7.2% of women reported being beaten, slapped or pinched during labor and 17.3% of women reported being tied down or restrained during labor. | Low quality |
| 63 | Ganle et al 2014 | Ashanti and Northern regions, Ghana | 185 pregnant and postpartum women  20 healthcare providers | Focus group discussions  Key informant interviews | Ghana’s free maternal healthcare policy appears to have increased the rate of facility-based childbirth. However, the maternal healthcare delivery system in Ghana lack many attributes of a functional healthcare system, including limited and unequal distribution of maternity services, poor quality of care, distrust in the healthcare system, difficulties relating to arranging suitable transportation to facilitate efficient referrals, women’s experiences of intimidation in healthcare facilities, unfriendly healthcare providers, cultural insensitivity, long waiting time before care is received, limited birthing choices, and lack of privacy at healthcare facilities. | High quality |
| 64 | Mirkuzie et al 2014 | Woreda 6 Gulele, Addis Ababa, Ethiopia | 19 migrant women who gave birth within the past year | In-depth interviews | Migrant women constitute disadvantaged communities in Addis Ababa and have unequal access to skilled birth care. Physical access to the health facility, social influence, maternal education, risk perception, perceived quality of care and disrespect were reported to be responsible for the disparities to access and utilize skilled birth care. The perceived quality of care at the health facility was reported as the most important factor for accessing and utilizing skilled care at birth. | High quality |
| 65 | Ng’anjo-Phiri 2014 | Kapiri Mposhi district, Zambia | 25 stakeholders (5 women with previous home birth, 5 husbands, 5 community leaders, 5 TBAs, 5 health providers)  Women attending ANC, women attending outpatient department | In-depth interviews with stakeholders  Focus group discussions with women attending ANC and/or outpatient department | Perceived quality of care and trust of the health system were important and influenced care-seeking behaviors. Safety, privacy and confidentiality during childbirth encouraged women to seek care at the health facility. However, poor accessibility to health facilities with skilled birth attendants, poor responsiveness of healthcare providers, unexpected costs of supplies and cultural values surrounding endurance at childbirth discouraged care seeking at birth. | Medium quality |
