# Supplementary material for: The Mistreatment of Women during Childbirth in Health Facilities Globally: A Mixed-Methods Systematic Review
Source: PLoS Med. 2015 Jun 30;12(6):e1001847. doi: 10.1371/journal.pmed.1001847 (PMC4488322; doi:10.1371/journal.pmed.1001847)
Supplement: S1 Text — Systematic review protocol outlining the background, objectives, and methodology of this systematic review. (DOCX) [file pmed.1001847.s007.docx]

**Disrespect and abuse during facility-based delivery:**

Systematic review protocol

Reviewers:

Meghan Bohren

Olha Lutsiv

Erin Hunter

Suprita Kudesia

Joao Paulo Souza

Contact:

Meghan Bohren, Consultant

Johns Hopkins Bloomberg School of Public Health

615 N Wolfe Street

Baltimore, MD 21205

mbohren@jhsph.edu

908-313-5262

Support provided by:

Department of Reproductive Health & Research

World Health Organization

USAID

**Background**

Although significant progress in reducing maternal mortality has been made since the establishment of the Millennium Development Goals (MDGs), around 800 women continue to die from puerperal causes around the world every day.^1^ Increasing the coverage of skilled birth assistance and institutional births is a central component of the strategy to reduce maternal mortality, and is also a key MDG-5 indicator.^2^ In many settings, however, women prefer to deliver outside of a health facility for a variety of reasons, including cost, distance to services, the perception of over-medicalized delivery practices, sociocultural influences, and perceptions of quality of care provided at health facilities.^3^ Previous literature has called for the systematic review of research to explore and synthesize research related to disrespect and abuse (D & A) by health workers during childbirth.^3^ Previous research also suggests that disrespectful and abusive treatment of women by health care providers and other staff in maternity units is a substantial issue and an impediment for expanding the coverage of institutional births.^4^ In order to strengthen the body of evidence on D & A in health facilities and to develop a strategy to remedy this situation, we will conduct a mixed-methods systematic review to determine what is known and where the knowledge gaps are. In particular, we aim to contribute to the definition of D & A through the development of a classification system, determine the prevalence of D & A (depending on available literature), and describe interventions designed to prevent and manage D & A.

***Definition***

Existing research on D & A during childbirth is mostly comprised of qualitative reports, and thus its definition is fairly broad and varies between studies. Thus far, a USAID landscape analysis has categorized such behaviors into seven groups: physical abuse, non-consented care, non-confidential care, non-dignified care, discrimination, abandonment of care, and detention in facilities.^3^ However, the landscape analysis does not describe their approach to developing the classification system and was not approached in a systematic fashion. This systematic review will utilize the definitions developed in the landscape analysis to assist in the development of a search strategy and as a starting point to move forward with this review. However, the reviewers will not use the landscape analysis to inform their evidence synthesis; rather, this review will maintain a systematic procedure of analyzing the findings and assessing the certainty of the findings. Developing a standard definition is essential for assessing the baseline prevalence of D & A in health facilities, for developing interventions aimed at preventing the occurrence of such behaviors, and for comparative purposes (between settings and over time).

Since the publication of the landscape analysis, there has been heightened interest and awareness concerning D & A during childbirth, which has stimulated new primary research. This review will add to the literature not only by updating the evidence on this topic, but it will also be the first systematic synthesis of the evidence. The rigorous methodology will afford us with more certainty that the definition and classification system of D & A during childbirth comprehensively considers studies across a variety of contexts.

***Prevalence***

An estimate of the prevalence of disrespectful delivery care is important to understand the scope and severity of the problem. Given that no standardized definition exists, prevalence estimates in the existing research are anticipated to be heterogeneous and poorly generalizable. Depending on the availability of prevalence estimates in the included studies, we will conduct a meta-analysis. If sufficient homogeneous data is not present to conduct a meta-analysis, then data will be presented by each study’s operational definition of D&A and corresponding prevalence.

1. **Objectives**

The objectives of this systematic review are to provide a comprehensive synthesis of the evidence on D & A in health facilities during childbirth. We aim to:

1. develop a standardized classification system for D & A in health facilities during childbirth;
2. identify the prevalence of D &A.
3. **Methodology**

*Inclusion and exclusion [SPICE]*

Setting

This review will include studies focusing on facility-based births, conducted in both rural and urban settings, without any restrictions on the country’s level of development (i.e., including low-, middle- and high-income countries). This review will include studies published in English, French, Portuguese, or Spanish.

Perspective

We seek the perspectives of any relevant players within the health system, including users, providers, and policy makers. Both female and male user perspectives will be included. Provider perspectives include, but are not limited to: physicians, nurses, nurse-midwives, midwives, traditional birth attendants, and doulas.

Phenomenon of interest

This review will explore the occurrence of D & A in health facilities by all levels of health workers (e.g., physicians, nurses, nurse-midwives, and midwives) and in all countries.

Comparison group

N/A

Experiences

The main outcomes of interest are user and provider experiences of D &A by health workers during childbirth in health facilities. This review will include quantitative, qualitative, and mixed-methods study designs.

Studies will be excluded if any of the following apply:

- Do not report on D & A of parturient women
- Not based on experiences during childbirth (i.e., ante-/postnatal)
- Not occurring in a health facility (i.e., community based)
- Do not explain the study methodology and process of data collection
- No primary data (i.e., secondary data analysis only or news articles)
- Language other than English, French, Portuguese, or Spanish
- Cannot find full text

*Screening, data extraction, and quality assessment*

Two reviewers will independently screen titles and abstracts identified in the search. Studies that appear to match the study inclusion criteria based on the screening of the titles and abstracts will then be reviewed in full by two independent reviewers, and the inclusion and exclusion criteria will be applied to determine if the study will be included in the analysis.

Data will be extracted using a standardized checklist (see Appendix A). Data will be collected on study design, methods, characteristics of participants, topics covered (e.g., prevalence of D &A, specific behaviors deemed to be D & A).

Two reviewers will independently assess study quality of all included studies using the CASP checklist (see Appendix B).

*Search strategy*

A search strategy has been developed by MB for PubMed (Appendix C), Embase (Appendix D) and CINAHL (Appendix E). The search will encompass all potentially relevant published and unpublished literature with no date restrictions. The search will be conducted through the utilization of the following databases:

- PubMed
- Embase
- CINAHL
- WHO Global Health Library
- Cochrane Library
- Google Scholar
- DARE
- CRD

Additional search strategies that may be utilized include the following:

1. “Snowballing” through references identified in articles captured by the initial database search;
2. Hand searching journals that frequently publish on topics of health care utilization (e.g., Health Education and Behavior, Social Sciences and Medicine, International Journal of Qualitative Methods, Journal of Advanced Nursing, and Qualitative Health Research);
3. Searching grey literature databases (e.g., OpenSIGLE [System for Information on Grey Literature]), for conference abstracts and other grey literature;
4. Personal contact with researchers in relevant fields of study

*Synthesis*

*Quantitative data*

Prevalence estimates of D & A during childbirth will likely be presented by individual study definition and reported prevalence. Due to the anticipated high heterogeneity between study definitions, designs, and populations, it is unlikely that the reviewers will be able to conduct a meta-analysis of prevalence data.

*Qualitative data*

Reviewers will employ thematic synthesis methodology, as discussed in Thomas & Harden (2008) and Bohren et al (in press), to illustrate the outcomes of the qualitative portion of the review.^5, 3^ Thematic synthesis is comprised of a three step process:

Stage 1 & 2 – coding and development of descriptive themes:

Enter the text findings verbatim into Atlas.ti or other qualitative software and independently code each line of text based on its content and meaning (e.g., axial coding). Codes will first be structured as “free” codes with no established link between them. As each study is coded, the reviewers will be able to translate concepts from one study to another. This will further develop the codebook, and new codes will be added as necessary. Reviewers will seek similarities and differences between the codes and group the codes according to a hierarchical structure. For example:

1.0-Passive disrespect and abuse

1.1-Abandonment/neglect

1.2-Non-consented care

1.3-Non-dignified care

1.4-Non-confidential care

1.5-Discrimination

2.0-Active disrespect and abuse

2.1-Physical abuse

2.2-Sexual abuse

2.3-Verbal abuse

In the first two stages, two reviewers will independently code the findings, then work as a team to generate analytical themes in stage 3.

Stage 3 – generating analytical themes:

In this stage, the reviewers will infer behaviors of D & A in health facility settings and conduct analytical discussions on these themes to develop a standardized definition of the phenomenon. This is a cyclical process and will be repeated until the themes generated are sufficiently conceptual to explain and describe the initial descriptive themes from stage 2.

Reviewers may use Atlas.ti throughout the stages of synthesis to assist in coding and analysis or may conduct the coding process by hand.

*Assessing the confidence in the findings*

The reviewers will assess the certainty of the qualitative findings utilizing the CerQual methodology and present the key findings in a summary of findings table.^3,6^

1. **Time frame**

15 September: Finalize review protocol, TIAB screening form, full text screening form, data extraction form, and search strategies

22 September: Conduct PubMed/Embase/CINAHL literature search; conduct grey literature search

6 October: Finish dual-reviewer title and abstract screening of identified studies

20 October: Finish full-text review of selected studies

8 November: Finish data extraction of included studies

17 November: Complete preliminary analyses of data for D & A meeting in Geneva

**19 November – 20 November: WHO consultation meeting on Disrespect & Abuse**

31 December – finish coding

15 February: Complete analyses and draft full report

28 February: Finalize full report for internal review

31 March: Finalize and submit manuscript to journal

1. **Plans for updating the review**

This review will be updated in 5 years from the publication date (anticipated 2020).

1. **Statement regarding conflict of interests**

None declared.

**References**

1. World Health Organization. Trends in Maternal Mortality: 1990-2010. WHO, UNICEF, UNFPA, & The World Bank. Geneva; WHO, 2012.
2. World Health Organization. Proportion of births attended by a skilled attendant: 2008 updates. Factsheet. Geneva; Department of Reproductive Health and Research, WHO, 2008.
3. Bohren M; Hunter E; Munthe-Kaas H; Souza JP; Vogel JP; Gulmezoglu M. Facilitators and barriers to facility-based delivery: A systematic review of qualitative evidence. Full report; Department of Reproductive Health and Research, WHO, 2013.
4. Bowser D & Hill K. Exploring evidence for disrespect and abuse in facility-based childbirth. Report of a Landscape Analysis. Washington, DC; USAID-TRACtion Project, 2010.
5. Thomas J & Harden A. Methods for the thematic synthesis of qualitative research in systematic reviews. BMC Medical Research Methodology: 2008; 8:45.
6. Glenton C, Colvin CJ., Carlsen B, Swartz A, Lewin S, Noyes J, Rashidian A. Barriers and facilitators to the implementation of lay health worker programmes to improve access to maternal and child health: qualitative evidence synthesis. Cochrane Database of Systematic Reviews 2013 In press.
